# Physical Activity, Body Composition, and Fitness Variables in Adolescents After Periods of Mandatory, Promoted or Nonmandatory, Nonpromoted Use of Step Tracker Mobile Apps: Randomized Controlled Trial

**DOI:** 10.2196/51206

**Published:** 2024-07-30

**Authors:** Adrián Mateo-Orcajada, Raquel Vaquero-Cristóbal, Jorge Mota, Lucía Abenza-Cano

**Affiliations:** 1 Facultad de Deporte Universidad Católica de Murcia Murcia Spain; 2 Research Group Movement Sciences and Sport (MS&SPORT), Department of Physical Activity and Sport Faculty of Sport Sciences University of Murcia Murcia Spain; 3 Faculdade de Desporto Universidade de Porto Porto Portugal

**Keywords:** body composition, detraining, new technologies, physical education subject, physical fitness, youth

## Abstract

**Background:**

It is not known whether an intervention made mandatory as a physical education (PE) class assignment and aimed at promoting physical activity (PA) in adolescents can create a healthy walking habit, which would allow further improvements to be achieved after the mandatory and promoted intervention has been completed.

**Objective:**

The aims of this study were to (1) investigate whether, after a period of using a step tracker mobile app made mandatory and promoted as a PE class assignment, adolescents continue to use it when its use is no longer mandatory and promoted; (2) determine whether there are changes in the PA level, body composition, and fitness of adolescents when the use of the app is mandatory and promoted and when it is neither mandatory nor promoted; and (3) analyze whether the covariates maturity status, gender, and specific app used can have an influence.

**Methods:**

A total of 357 students in compulsory secondary education (age: mean 13.92, SD 1.91 y) participated in the study. A randomized controlled trial was conducted consisting of 2 consecutive 10-week interventions. Participants’ PA level, body composition, and fitness were measured at baseline (T1), after 10 weeks of mandatory and promoted app use (T2), and after 10 weeks of nonmandatory and nonpromoted app use (T3). Each participant in the experimental group (EG) used 1 of 4 selected step tracker mobile apps after school hours.

**Results:**

The results showed that when the use of the apps was neither mandatory nor promoted as a PE class assignment, only a few adolescents (18/216, 8.3%) continued the walking practice. After the mandatory and promoted intervention period (T1 vs T2), a decrease in the sum of 3 skinfolds (mean difference [MD] 1.679; *P*=.02) as well as improvements in the PA level (MD –0.170; *P*<.001), maximal oxygen uptake (MD –1.006; *P*<.001), countermovement jump test (MD –1.337; *P*=.04), curl-up test (MD –3.791; *P*<.001), and push-up test (MD –1.920; *P*<.001) in the EG were recorded. However, the changes between T1 and T2 were significantly greater in the EG than in the control group only in the PA level and curl-up test. Thus, when comparing the measurements taken between T1 and T3, no significant changes in body composition (*P*=.07) or fitness (*P*=.84) were observed between the EG and the control group. The covariates maturity status, gender, and specific app used showed a significant effect in most of the analyses performed.

**Conclusions:**

A period of mandatory and promoted use of step tracker mobile apps benefited the variables of body composition and fitness in adolescents but did not create a healthy walking habit in this population; therefore, when the use of these apps ceased to be mandatory and promoted, the effects obtained disappeared.

**Trial Registration:**

ClinicalTrials.gov NCT06164041; https://clinicaltrials.gov/study/NCT06164041

## Introduction

### Background

In recent years, a growing significance has been placed on the engagement of adolescents in physical activity (PA), given the decrease in active time and the increase in sedentary activities and screen time [1]. These behaviors have negatively impacted the health of adolescents by increasing the risk of cardiovascular disease and other associated chronic diseases [2]. This situation has prompted the implementation of new practices that have been shown to be effective in increasing the level of sports practice in adolescents [3,4]. In this context, interventions that incorporate electronic devices have gained relevance [5], given their extensive use during the COVID-19 pandemic and their integration into the daily lives of adolescents [6].

Mobile sports apps have emerged as valuable resources in promoting PA among adolescents [7], and it has been observed that interventions with mobile devices have made it possible to increase moderate-intensity PA and daily step count among users [8]. This has also had a positive impact on the health of the adolescent population because the use of these mobile apps has improved their body composition and fitness levels [9,10], which are fundamental for their subsequent development [11,12]. This is because adolescents who are overweight or obese have a high probability of remaining so in adulthood [13], with the associated high health risk for cardiovascular and respiratory diseases [14]. On the contrary, adequate levels of body fat, within the limits considered healthy [15], as well as an adequate physical fitness level, especially good cardiorespiratory fitness, are indicators of adequate health and serve as preventive factors against various diseases in adulthood [16].

In this regard, mobile apps seem to be effective tools for improving the health status of adolescents and for preventing future health risks. It is crucial to emphasize that the effectiveness of increasing adolescents’ PA level through mobile apps was evident only in studies in which the use of these apps was mandatory. Specifically, the promotion of app use as an assignment in physical education (PE) classes played a significant role in achieving positive outcomes [9,17]. Furthermore, it is worth noting that no major differences were found in the effects achieved by the intervention when comparing the different mobile apps used, as long as they were all step trackers [9]; however, the gender of the adolescents was shown to be a determinant factor in the benefits obtained because female adolescents used these apps more often than male adolescents during the mandatory and promoted intervention period, which led to significant differences in the benefits obtained in BMI, corrected calf girth, fat mass, and physical fitness [18]. Therefore, the mandatory use of apps seemed to be effective in this population, although it should be noted that a previous study showed that the first weeks of the intervention were the most effective in the adolescent population due to the novelty of the intervention, but as the intervention progressed, the effects were reduced [19]. This is a relevant aspect because there is a considerable loss of adherence after the first weeks of the intervention [8], which could negatively influence the overall benefits obtained.

In addition to the loss of adherence, there are also periods in the school calendar when PA decreases, such as holiday breaks. These are characterized by the absence of students from school, which makes it difficult to promote the use of this type of intervention as a PE class assignment. This has a particular significance because prior research has revealed a detraining effect, wherein the gains in body composition and fitness achieved during an aerobic intervention period were subsequently lost, leading to a regression to preintervention levels [20].

Therefore, it is essential to verify whether, after a period of mandatory and promoted use of step tracker mobile apps as a PE class assignment, which has shown beneficial effects on body composition and physical fitness in previous research [9], it is possible to create a healthy walking habit in adolescents and to have them continue using the apps when they are neither mandatory nor promoted to try to avoid detraining effects. This would allow us to define strategies to compensate for the loss of adherence and decrease in PA level observed during holiday periods, similar to previous research on flexibility in adolescents [21]. However, no previous research is known to have analyzed whether the effects achieved throughout a period of mandatory and promoted use of step tracker mobile apps are maintained over time when their use is neither mandatory nor promoted as a PE class assignment. Furthermore, previous studies in this area have not analyzed whether the effects of such interventions may depend on the maturity status of adolescents, although numerous studies have shown that the rate of maturation during adolescence varies between individuals [22]. Thus, this factor may condition the changes in body composition and fitness variables in adolescents [23].

### Objectives

Therefore, considering the absence of previous research analyzing whether adolescents aged 12 to 16 years continue their walking practice with step tracker mobile apps when their use is no longer mandatory and promoted as a PE class assignment, as well as the influence that covariates such as maturity status, gender, and specific app used may have on the results, the aims of this study were to (1) investigate whether, after a period of mandatory and promoted use of a step tracker mobile app as a PE class assignment, adolescents continue to use it when its use is no longer mandatory and promoted; (2) determine whether there are changes in PA level, body composition, and fitness of adolescents when the use of the app is mandatory and promoted as a PE class assignment, as well as whether maturity status, gender, and specific app used can have an influence on the results; and (3) analyze whether there are changes in PA level, body composition, and fitness of adolescents when the use of the app is neither mandatory nor promoted as a PE class assignment, as well as whether maturity status, gender, and specific app used can have an influence on the results.

### Hypotheses

On the basis of the aims of the research and previous research involving technological devices (eg, wearables) or websites, it is hypothesized that adolescents will stop using the mobile app during the period of nonmandatory and nonpromoted use (H1); that there will be significant differences in PA level, body composition, and fitness of adolescents during the mandatory and promoted period, influenced by maturity status and gender but not by specific app used (H2); and that some of the benefits achieved by adolescents during the mandatory and promoted period will be lost after the mandatory and promoted intervention has been completed, with the results being influenced by maturity status and gender but not by specific app used (H3).

## Methods

### Design

**Figure 1:**
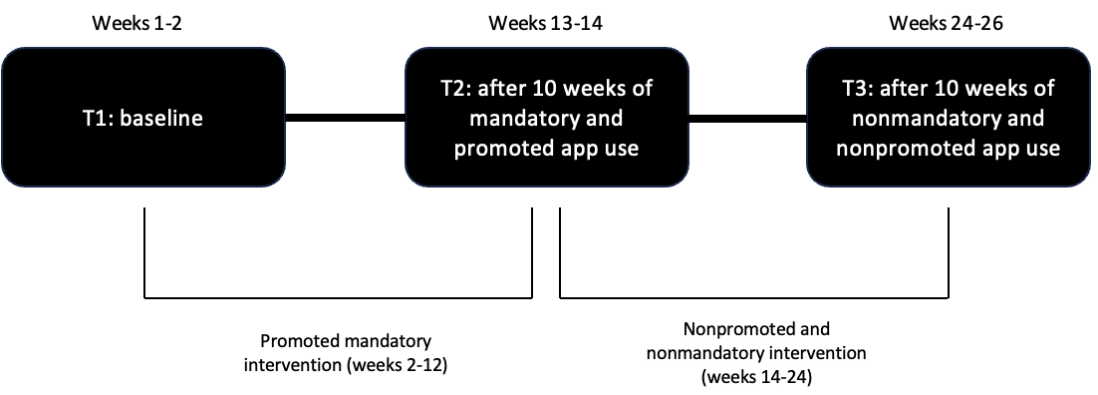
Timeline of the study.

The intervention in this study was carried out by replicating the methodology of previous research [9], the main difference being that this research analyzed what happens to the study variables when the mandatory intervention ends and becomes a nonmandatory, nonpromoted intervention. Our new research design comprised 3 data collection periods (T1: baseline, T2: after 10 weeks of mandatory and promoted app use, and T3: after 10 weeks of nonmandatory and nonpromoted app use) with a total duration of 26 weeks. T1 took place in the first 2 weeks (weeks 1-2); the mandatory intervention with the step tracker mobile apps promoted as a PE class assignment took place in the following 10 weeks (weeks 2-12); T2 took place in the next 2 weeks (weeks 13-14); the use of the step tracker mobile apps was neither mandatory nor promoted during the following 10 weeks (weeks 14-24); and T3 took place in the last 2 weeks of the study (weeks 24-26). Figure 1 shows the timeline of the study. The intervention began on January 9, 2023, and ended on June 23, 2023.

This study was a randomized controlled trial. It followed the CONSORT (Consolidated Standards of Reporting Trials) guidelines [24] and was preregistered at ClinicalTrials.gov (NCT06164041). A convenience sampling method was used to recruit adolescents from accessible educational institutions.

The study used a specific research model (Table 1).

**Table 1:** Research model.

Variable type	Construct	Groups included	Variables included
Independent	Physical activity level	App use group and control group	Subjective assessment of the level of physical activity
Dependent	Kinanthropometric and body composition variables	App use group and control group	Body mass; height; BMI; sitting height; sum of 3 skinfolds; corrected arm, thigh, and calf girths; waist girth; hips girth; waist to hip ratio; muscle mass; and fat mass
Dependent	Physical fitness variables	App use group and control group	VO_2max_^a^, CMJ^b^ test, curl-up test, and push-up test
Covariates	Variables	App use group and control group	Maturity status, gender, and specific app used

^a^VO_2max_: maximal oxygen uptake.

^b^CMJ: countermovement jump.

### Ethical Considerations

This study was approved by the institutional ethics committee of the Catholic University of Murcia (code CE022102) and adhered to the guidelines set forth by the World Medical Association and the Declaration of Helsinki. Adolescents who expressed willingness to participate in the study were required to sign an informed consent form, with both adolescents and their parents acknowledging their understanding of the study aims and procedures.

### Participants

We recruited participants from 2 compulsory secondary schools located in Murcia. These schools were chosen because of their large student population in secondary education within their respective localities. Initially, the research team contacted the schools to provide a detailed explanation of the study’s procedure and objectives. If a particular school declined to participate, the school with the next largest number of students in the locality was approached. Once the school’s approval was obtained, the PE department heads were contacted. Subsequently, a face-to-face meeting was arranged with interested students and their parents to discuss the study further.

The minimum sample size necessary for the study was calculated using RStudio software (version 3.15.0; Posit Software PBC) and followed the methodology used in previous studies [25], in which the SD value (0.64) from previous studies that used a similar design with 3 data points to measure changes in PA among adolescents was used [26]. With an estimated error (d) of 0.067 and a CI of 95%, the required sample size was determined to be 350 adolescents.

**Figure 2:**
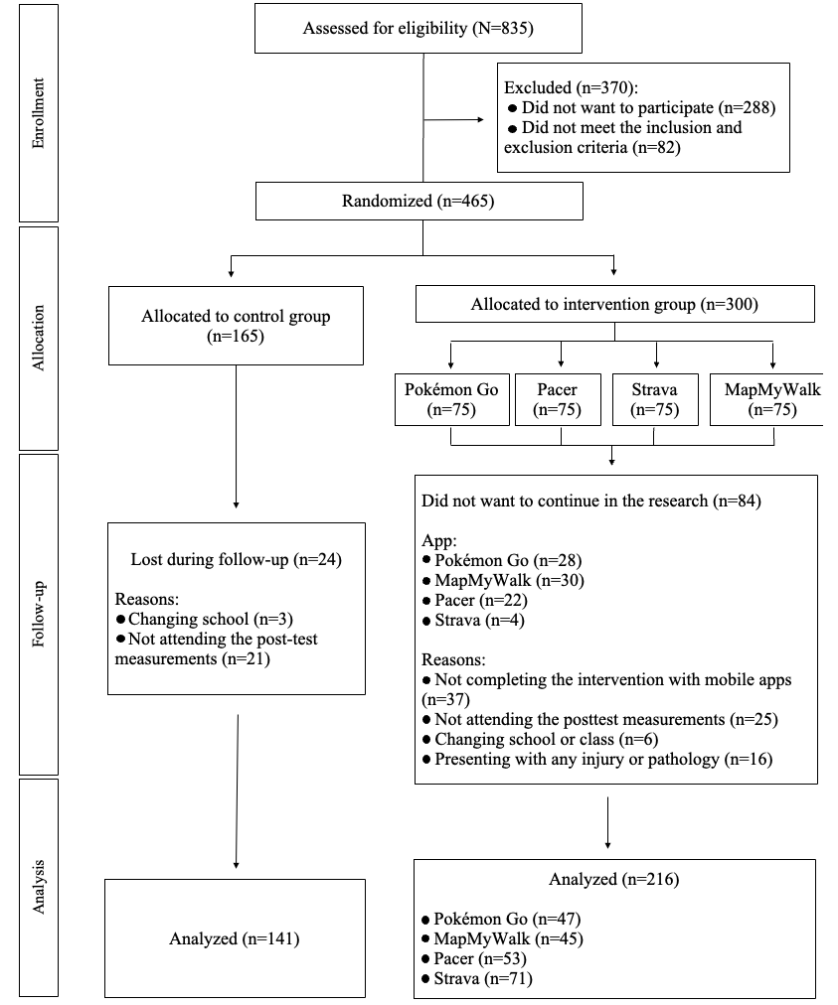
Sample selection flowchart.

Figure 2 illustrates the flowchart for the selection of the sample. The final sample comprised 357 adolescents aged between 12 and 16 years. The participants were assigned to the different groups using a cluster randomized design [27]. Group assignment was concealed from the researcher who analyzed each participant’s compliance with the inclusion and exclusion criteria. The inclusion criteria for the study were as follows: (1) enrollment in 1 of the selected educational institutions, (2) aged between 12 and 16 years, (3) completion of all questionnaires and physical tests during the 3 measurement periods (T1, T2, and T3), (4) attending the kinanthropometric and body composition assessment sessions, and (5) absence of any pathology or injury that would hinder participation in the tests or measurements conducted. The exclusion criteria were as follows: (1) missing >20% of the mandatory PE sessions throughout the academic year, (2) lack of a mobile phone, (3) failure to meet the minimum mandatory weekly distance requirement in the experimental group (EG) when app use was mandatory and promoted, (4) changing schools or class group during the course of the intervention, (5) starting or ending any form of PA (for reasons unrelated to the study) during the intervention that could alter the PA level being assessed as part of the study, and (6) having presented with any illness during the follow-up period that would have prevented the participant from engaging in their usual PA.

### Randomization and Blinding

After the recruitment and selection of the adolescents, meetings were held with the teachers to provide them with a clear understanding of the trial’s purpose and the randomization process. Parents or legal guardians of the potential participants at each school were notified through a letter that explained the study’s objectives and procedures. The principal investigator, along with other uninvolved investigators, carried out the randomization process using a computer-generated random number table. The randomization assigned all students within the same class at each school to the same mobile app group. The classes were randomly assigned to participate as intervention or control classes. A total of 16 classes were finally randomized, of which 11 (69%) were included in the EG and 5 (31%) in the control group (CG). The ratio chosen for the randomized clusters was 2:1 (for every 2 classes included in the EG, 1 was included in the CG) because previous research with mobile apps has highlighted the lack of adherence to mobile apps, and we wanted to ensure that we had enough participants in the EG to account for a possible high dropout rate (close to 35%) from hindering the extrapolation of the results [28]. The control classes were instructed to continue their regular PE classes, while the intervention was offered to them after the final data collection took place. Baseline measurements were taken before the randomization process. All measurers were blinded to the group to which each individual belonged during the second and third measurements, as well as to the individual’s ratings in the previous measurements.

### Instruments

The instruments used in this study were the same as those used in previous investigations [9] because these are valid and reliable in the adolescent population.

#### Questionnaire Measurement

A sociodemographic questionnaire developed ad hoc was administered to obtain data on the age and gender of the participants, their regular PA, and the occurrence of injury or illness, following the pattern of previous studies [23].

PA level was measured using the Physical Activity Questionnaire for Adolescents (PAQ-A) [29]. This questionnaire had been previously validated in Spanish and showed satisfactory reliability, with an intraclass correlation coefficient of 0.71 for the final score [30].

#### Kinanthropometric and Body Composition Measurement

The anthropometric measurement included 3 basic parameters (body mass, height, and sitting height), 3 skinfold measurements (triceps, thigh, and calf), and 5 girth measurements (arm relaxed, waist, hips, thigh, and calf) [31]. Measurements were performed by anthropometrists (level 3 and level 4) accredited by the International Society for the Advancement of Kinanthropometry [31].

The anthropometric instruments used were the same as those used in previous research [9]: a Harpenden skinfold caliper, a Lufkin W606PM anthropometric tape measure, a Tanita BC418-MA segmental scale, and a Seca 213 stadiometer. All instruments were calibrated before the beginning of each of the measurements (T1, T2, and T3).

The following derived variables were calculated from the anthropometric measurements: BMI, muscle mass [32], fat mass [33], sum of 3 skinfolds (triceps, thigh, and calf), waist to hip ratio (waist girth/hips girth), and corrected girths of the arm (arm relaxed girth – [π × triceps skinfold]), thigh (middle thigh girth – [π × thigh skinfold]), and calf (calf girth – [π × calf skinfold]) [34]. The body composition formulas have been used in previous research [35] and are the ones most often recommended for evaluation in this population [36].

The maturity offset was calculated according to the procedure established by Mirwald et al [37] and using gender-specific formulas: –9.37 + 0.0001882 × ((height – sitting height) × sitting height) – 0.0022 × (age × (height – sitting height)) + 0.005841 × (age × sitting height) – 0.002658 × (age × weight) + 0.07693 × (weight / height). The result of the maturity offset equation is expressed in years from the age at peak height velocity (PHV) when the result is positive and in years to the age at PHV when the result is negative.

The same anthropometrist performed the T1, T2, and T3 measurements on each participant to reduce interevaluator error. The intra- and interevaluator technical errors of measurement [34] were 0.02% and 0.04% for basic measurements, 1.09% and 1.87% for skinfolds, and 0.03% and 0.08% for girths. The correlation coefficients of the anthropometrists with respect to a level 4 expert anthropometrist were 0.96 for basic measurements, 0.91 for skinfolds, and 0.93 for girths.

#### Physical Fitness Measurements

Cardiorespiratory fitness was evaluated using the 20-meter shuttle run test. The test ends when the adolescent is unable to complete the required distance in the indicated time twice consecutively or when he or she reaches exhaustion. Upon completion of the test, the final speed at which the adolescent concluded the shuttle run was used to calculate their maximal oxygen uptake (VO_2max_) [38]. This test has high validity and reliability for the determination of VO_2max_ [39].

Lower limb explosive strength was assessed by means of the countermovement jump (CMJ). Adolescents had to perform a 90-degree knee flexion at maximum speed, keeping the back fully straight with hands placed on the hips, followed by a maximal knee extension to jump [40]. The adolescents were required to execute a maximal jump while maintaining their hands on their hips throughout the test. The jump height was determined by measuring the flight height achieved during the jump [40].

For the measurement of abdominal strength and endurance, we used the curl-up test. For the execution to be valid, the adolescents had to keep their feet fully supported on the floor and their arms crossed on the chest, and the trunk flexion had to allow the upper back to be lifted off the floor [41]. The test ended when the time was up (1 min) or when the participant reached exhaustion.

The push-up test was used to evaluate upper body strength. The repetition was valid if the adolescents managed to fully extend their arms and return to the 90-degree position [42]. The adolescents had to perform as many push-ups as possible in 1 minute. The test ended when the time was up (1 min) or when the participant reached exhaustion.

### Procedure

The procedure used was also similar to that used in previous research [9], but the difference was that in this study, the intention was to discover how adherence to the intervention changed when the intervention was no longer mandatory or promoted as a PE class assignment and how this affected the variables analyzed. Therefore, unlike previous studies [9], this study comprised 3 measurement periods (T1, T2, and T3), with 2 interventions carried out consecutively. The first one was mandatory and promoted as a PE class assignment, and the second one was neither promoted nor promoted as a PE class assignment.

The data collection process followed the same protocol as in previous research [9,43], with the sociodemographic and PAQ-A questionnaires completed first, followed by the anthropometric measurements. Once these were completed, the fitness tests were explained and performed randomly, twice each, with the 20-meter shuttle run test performed last and only once. The physical test protocol adhered to the guidelines set forth by the National Strength and Conditioning Association, with the aim of minimizing interference between tests and allowing sufficient recovery time from the exertion and metabolic demands of the assessments [44].

To minimize bias in the measurements, they were carried out under the same conditions for all students. The PE class hour was used for the measurements and the adolescents were always measured at the same time and on the same day of the week at T1-T2-T3. The questionnaires were completed in a reserved space in which the adolescents did not have any distractions that could have conditioned their answers. In addition, while the researchers resolved any possible doubts, in no case did they condition the adolescents’ responses. For the anthropometric measurements, the air-conditioned locker rooms of the sports pavilion were used to minimize variability due to temperature and humidity fluctuations across the 3 measurement periods. To conduct the physical tests, the indoor sports pavilion at each school was used, which was specifically chosen to eliminate the influence of atmospheric variables that could potentially affect the results and introduce bias.

### Mobile App Intervention

Before starting the intervention, 465 adolescents participated in pretest measurements (T1; Figure 2). The mandatory and promoted intervention lasted 10 weeks, during which the adolescents were required to use 1 of the 4 selected apps: Pokémon Go, Pacer, Strava, or MapMyWalk. These apps were selected based on their implementation of a substantial number of behavior change techniques [45] specifically designed to effectively enhance PA level among users. Moreover, they have already been used in previous research with adolescents, with participants demonstrating good adherence [9]. The assignment to each of the app groups was randomized by class group. Thus, initially, an equal number of adolescents was assigned to use each app (Pokémon Go: 75/300, 25%; MapMyWalk: 75/300, 25%; Pacer: 75/300, 25%; and Strava: 75/300, 25%). Of the 465 adolescents, 165 (35.5%) were assigned to the CG.

Before starting the mandatory and promoted intervention, the adolescents were provided with instructions on the proper use of step tracker mobile apps. The aim of the first phase was for students, after receiving instructions on the correct use of the apps, to use them in a manner guided by the PE teachers so that they could become familiar with their use and interface. For this purpose, after randomization, a meeting was held with each of the class groups that were assigned to the EG. In this meeting, the students installed the app corresponding to their class group, and an explanation on the functioning of each was provided to them. Any doubts were resolved by the researchers and the PE teachers. The researchers in charge of explaining how the apps worked were not involved in the measurements or subsequent analysis because they knew which student belonged to each app group and which student belonged to the CG. Once each app had been described and its use explained, a training plan was drawn up to be followed during the period of mandatory and promoted use. During the initial week, the adolescents were instructed to achieve a minimum of 5000 steps or cover a distance of at least 3.19 km each time they used the app. It was established that approximately 1565 steps equals 1 km [46]. This minimum distance was defined to ensure that the adolescents exceeded the sedentary threshold [47]. The initial distance was progressively increased weekly until reaching a distance of 15,520 steps or 8 km each time they used the app. In addition, the researchers followed up with the PE teachers to ensure that the distance was completed by the students every week.

The adolescents were motivated to use the app for a duration of 10 weeks, aiming for a minimum use of 3 times per week. This frequency aligned with the PA recommendations set forth by the World Health Organization [48]. The duration of 10 weeks was justified based on previous research with adolescents, in which a short or moderate duration (6-12 wk) was more effective for producing changes than a longer duration [19], and to be able to adjust it to the duration of the academic year. To encourage the use of the mobile apps during the period of mandatory and promoted use, PE teachers rewarded participation in the study with up to 1 point in the final PE grade for those who completed the study.

After the mandatory and promoted intervention with the mobile apps, posttest 1 measurements were carried out (T2). This was followed by a 10-week period in which the use of the apps was no longer promoted or mandatory as a PE class assignment, after which posttest 2 (T3) measurements were taken. During this period of nonmandatory and nonpromoted use, the adolescents could continue to use the mobile apps voluntarily, just as they would in their daily lives. The adolescents who continued to use the apps were recorded.

In both the mandatory and promoted and the nonmandatory and nonpromoted periods, a researcher who did not participate in the data collection process recorded the distance (in kilometers) and the number of steps taken by each participant after using the mobile apps daily.

A total of 357 adolescents participated in the final measurements (Pokémon Go: n=47, 13.2%; MapMyWalk: n=45, 12.6%; Pacer: n=53, 14.8%; Strava: n=71, 19.9%; and CG: n=141, 39.5%), while 108 adolescents dropped out of the program (Pokémon Go: n=28, 25.9%; MapMyWalk: n=30, 27.8%; Pacer: n=22, 20.4%; Strava: n=4, 3.7%; and CG: n=24, 22.2%; Figure 2). Adolescents who, despite the mandatory and promoted use, did not start using the mobile app were considered to have dropped out, as were those who did not complete at least 25% of the total training volume required because previous research has shown that this is the minimum volume needed to produce significant changes in body composition and fitness variables [43]. Adolescents who completed at least 25% of the training volume required were retained in their respective app groups, those who exceeded 25% of the required training volume but did not complete the entire intervention received up to half a point in the final PE grade, and those who dropped out or did not complete at least 25% of the training volume did not receive any bonus point in the final PE grade.

### Data Analysis

The normality of the data was assessed using the Kolmogorov-Smirnov test, alongside analyses of skewness, kurtosis, and variance. As the variables exhibited a normal distribution, parametric tests were used for their analysis. Three repeated measures ANOVAs were performed. On the first, the group factor was used as the grouping variable; on the second, the time point factor was used; and on the third, the differences in the changes between the CG and EG at the different time points were assessed. In this way, intra- and intergroup differences were determined for each of the study variables. A subsequent Bonferroni analysis made it possible to determine the statistical differences between each of the pairs compared. Three analyses of covariance were also performed to determine the influence of the covariates maturity status, gender, and specific app used on the results obtained for the study variables. Effect size was analyzed using partial eta-squared (η_p_²) and was defined as small (≥0.10), moderate (≥0.30), large (≥0.50), very large (≥0.70) or extremely large (≥0.90). These translate into 0.20, 0.60, 1.20, 2.0 and 4.0 for standardized differences in means [49]. A *P* value <.05 was used to establish statistical significance. The data analysis was performed using SPSS software (version 25.0; IBM Corp).

## Results

### Overview

Of the 357 adolescents, 186 (52.1%) were male, and 171 (47.9%) were female. Of the 186 male adolescents, 26 (14%) used Pokémon Go, 35 (18.8%) used Strava, 29 (15.6%) used Pacer, 25 (13.4%) used MapMyWalk, and 71 (19.9%) were in the CG. Of the 171 female adolescents, 21 (12.3%) used Pokémon Go, 36 (21.1%) used Strava, 24 (14%) used Pacer, 20 (11.7%) used MapMyWalk, and 70 (40.9%) were in the CG. The mean age of the male adolescents was 13.91 (SD 1.22) years, with a mean maturity offset of 0.20 (SD 1.39) years. Their mean body mass was 55.68 (SD 13.09) kg, and their mean height was 164.59 (SD 10.07) cm. The mean age of the female adolescents was 13.89 (SD 1.21) years, with a mean maturity offset of 1.50 (SD 0.90) years. Their mean body mass was 52.53 (SD 10.92) kg, and their mean height was 158.76 (SD 6.32) cm.

Of the 216 adolescents in the EG during the period of mandatory and promoted use of the app, only 18 (8.3%) continued to use the apps independently during the nonmandatory and nonpromoted period. The average distance walked by these adolescents was 47.69 (SD 23.80; range 5-200) km in the 10 weeks of nonpromoted and nonmandatory use.

### Differences in the EG and CG at the Different Study Time Points

Table 2 shows the differences in the measurements taken in the EG and CG at the different study time points (T1, T2, and T3). Significant differences in the PA level were observed exclusively within the EG: it was higher at T2 than at T1 (*P*<.001) but lower at T3 than at T2 (*P*=.03). No differences were found between T1 and T3 (*P*=.47) either in the EG or in the CG in any of the comparisons. In terms of the anthropometric and body composition variables, body mass and height significantly increased in both EG and CG between T1 and T2 (*P*<.001) and T1 and T3 (*P*=.002-.008), but no differences were found between T2 and T3 (*P*=.23-.99). In the sum of 3 skinfolds, the EG showed a significant decrease between T1 and T2 (*P*=.02), but a significant increase was found between T2 and T3 (*P*=.03). All corrected girth (*P*<.001-.049) and muscle mass (*P*<.001-.007) measurements showed significant increases in both groups between the 3 time points, including hips girth (*P*<.001-.03). By contrast, BMI (*P*=.01-.99), sitting height (*P*=.11-.99), fat mass (*P*=.07-.99), and waist girth (*P*=.23-.99) did not show differences in any of the groups in any of the comparisons.

**Table 2:** Differences in the experimental group (EG) and the control group (CG) during the different study time points (intragroup differences).

Variable and group		T1, mean (SD)	T2, mean (SD)	T3, mean (SD)	Mean difference (T1–T2)	*P* value	Mean difference (T1–T3)	*P* value	Mean difference (T2–T3)	*P* value	*F* test (*df*)	η_p_²
**Subjective level of physical activity**												
	EG	2.62 (0.68)	2.79 (0.59)	2.68 (0.68)	–0.170	<.001	–0.060	.47	0.110	.03	11.208 (1)	0.060
	CG	2.72 (0.64)	2.72 (0.73)	2.66 (0.71)	–0.004	.99	0.057	.79	0.061	.74	0.791 (1)	0.004
**Body mass (kg)**												
	EG	55.16 (12.87)	56.06 (12.69)	56.03 (11.63)	–0.901	<.001	–0.873	.004	0.028	.99	24.833 (1)	0.126
	CG	52.56 (10.84)	53.51 (10.71)	53.71 (10.72)	–0.946	<.001	–1.148	.002	–0.202	.99	18.259 (1)	0.098
**Height (cm)**												
	EG	162.35 (SD 9.04)	163.11 (8.98)	163.27 (9.74)	–0.760	<.001	–0.915	.008	–0.155	.99	20.640 (1)	0.107
	CG	161.02 (8.82)	161.63 (8.77)	162.25 (9.31)	–0.613	<.001	–1.229	.003	–0.616	.23	10.789 (1)	0.059
**BMI (kg/m^2^)**												
	EG	20.87 (3.84)	20.98 (3.69)	20.93 (3.69)	–0.107	.09	–0.051	.99	0.055	.53	2.688 (1)	0.015
	CG	20.19 (3.34)	20.44 (3.21)	20.26 (3.19)	–0.251	.11	–0.071	.91	0.179	.40	12.041 (1)	0.066
**Sitting height (cm)**												
	EG	84.75 (9.59)	85.54 (4.78)	82.90 (19.43)	–0.799	.98	1.846	.40	2.644	.11	2.184 (1)	0.012
	CG	82.85 (12.28)	83.43 (11.18)	83.12 (15.32)	–0.580	.99	–0.263	.99	0.317	.99	0.170 (1)	0.001
**Sum of 3 skinfolds (mm)**												
	EG	52.03 (26.58)	50.35 (24.51)	51.50 (25.40)	1.679	.02	0.530	.99	–1.149	.32	5.599 (1)	0.032
	CG	45.05 (24.18)	44.44 (23.30)	45.12 (23.80)	0.607	.99	–0.072	.99	–0.680	.66	0.889 (1)	0.005
**Corrected arm girth (cm)**												
	EG	20.83 (2.77)	21.26 (2.79)	21.48 (2.83)	–0.431	<.001	–0.651	<.001	–0.220	<.001	52.455 (1)	0.235
	CG	20.81 (2.75)	21.20 (2.67)	21.49 (2.67)	–0.388	<.001	–0.676	<.001	–0.288	<.001	36.041 (1)	0.174
**Corrected thigh girth (cm)**												
	EG	39.18 (4.78)	40.11 (4.57)	40.19 (4.73)	–0.924	<.001	–1.010	<.001	–0.086	.99	17.816 (1)	0.111
	CG	39.43 (5.24)	39.89 (4.22)	40.64 (4.40)	–0.454	.049	–1.201	<.001	–0.747	<.001	14.503 (1)	0.078
**Corrected calf girth (cm)**												
	EG	28.95 (3.55)	29.27 (2.91)	29.35 (2.90)	–0.321	.049	–0.400	.01	–0.078	.45	4.271 (1)	0.024
	CG	28.75 (2.75)	29.28 (2.66)	29.37 (2.68)	–0.531	.005	–0.621	.001	–0.090	.53	6.510 (1)	0.037
**Waist girth (cm)**												
	EG	68.36 (8.84)	68.44 (8.44)	68.48 (9.05)	–0.083	.99	–0.121	.99	–0.038	.99	0.196 (1)	0.001
	CG	67.57 (7.13)	67.84 (7.24)	68.01 (7.49)	–0.276	.45	–0.443	.23	–0.167	.99	1.644 (1)	0.010
**Hips girth (cm)**												
	EG	89.24 (9.25)	90.18 (8.74)	90.55 (8.77)	–0.937	<.001	–1.313	<.001	–0.377	.03	21.776 (1)	0.113
	CG	86.40 (7.88)	87.67 (7.83)	88.31 (7.96)	–1.270	<.001	–1.906	<.001	–0.636	.001	29.078 (1)	0.145
**Waist to hip ratio**												
	EG	0.77 (0.05)	0.76 (0.05)	0.76 (0.06)	0.007	<.001	0.010	<.001	0.003	.38	16.446 (1)	0.088
	CG	0.78 (0.05)	0.77 (0.05)	0.77 (0.06)	0.008	<.001	0.012	<.001	0.004	.41	15.093 (1)	0.081
**Muscle mass (kg)**												
	EG	17.91 (5.05)	18.60 (5.11)	18.85 (5.18)	–0.694	<.001	–0.942	<.001	–0.248	.007	37.598 (1)	0.181
	CG	18.38 (4.74)	18.85 (4.48)	19.39 (4.65)	–0.469	<.001	–1.009	<.001	–0.541	<.001	26.987 (1)	0.137
**Fat mass (%)**												
	EG	22.73 (10.23)	22.20 (9.84)	22.31 (9.90)	0.530	.07	0.425	.26	–0.105	.99	2.544 (1)	0.015
	CG	20.10 (10.12)	19.79 (9.90)	19.89 (9.75)	0.314	.84	0.210	.99	–0.104	.99	0.620 (1)	0.004
**VO_2max_^a^ (ml/kg/min)**												
	EG	38.03 (4.89)	39.03 (5.71)	38.12 (6.69)	–1.006	<.001	–0.094	.99	0.913	.005	12.772 (1)	0.077Yes
	CG	38.76 (5.10)	39.39 (5.10)	39.16 (6.49)	–0.632	.06	–0.404	.89	0.228	.99	2.780 (1)	0.018
**CMJ^b^ test (cm)**												
	EG	21.82 (7.53)	23.16 (7.93)	23.19 (8.16)	–1.337	.04	–1.371	.03	–0.034	.99	4.234 (1)	0.023
	CG	22.40 (7.01)	22.97 (9.26)	24.56 (8.60)	–0.572	.99	–2.163	.03	–1.591	.03	6.259 (1)	0.034
**Curl-up test (repetitions, n)**												
	EG	20.51 (11.49)	24.31 (10.69)	24.80 (11.28)	–3.791	<.001	–4.282	<.001	–0.490	.99	22.022 (1)	0.115
	CG	20.99 (11.10)	22.53 (12.26)	24.07 (11.71)	–1.540	.28	–3.073	.001	–1.533	.16	7.351 (1)	0.041
**Push-up test (repetitions, n)**												
	EG	6.80 (9.41)	8.72 (10.95)	7.93 (10.45)	–1.920	<.001	–1.128	.01	0.793	.34	9.829 (1)	0.061
	CG	7.64 (9.24)	8.62 (9.66)	8.36 (10.27)	–0.975	.28	–0.717	.44	0.258	.99	1.868 (1)	0.012

^a^VO_2max_: maximal oxygen uptake.

^b^CMJ: countermovement jump.

Regarding physical fitness, VO_2max_ significantly increased in the EG between T1 and T2 (*P*<.001), but it significantly decreased between T2 and T3 (*P*=.005). The CG exhibited no discernible differences. The CMJ score significantly increased at T2 compared to T1 (*P*=.04) and remained elevated at T3 compared to T1 (*P*=.03). In the CG, the adolescents showed a higher score at T3 than at T1 (*P*=.003) and T2 (*P*=.03). The curl-up test showed a significant increase in the EG between T1 and T2 (*P*<.001), which remained the same at T3 (*P*<.001), while in the CG, the increase was smaller and was only observed between T3 and T1 (*P*=.001). Finally, in the push-up test, an increase was observed in the score between T1 and T2 (*P*<.001), which remained, although it was less pronounced, at T3 (*P*=.01) only in the EG (Table 2).

As shown in Multimedia Appendix 1, the covariate maturity status was a determinant factor in the differences found in the EG for the variables PA level, body mass, height, sum of 3 skinfolds, corrected girths, hips girth, waist to hip ratio, muscle mass, VO_2max_, CMJ test, curl-up test, and push-up test between T1 and T2 (*P*<.001-.04); for the variables PA level, sum of 3 skinfolds, corrected arm girth, hips girth, muscle mass, and VO_2max_ between T2 and T3 (*P*<.001-.03); and for the variables height, corrected girths, hips girth, waist to hip ratio, muscle mass, CMJ test, curl-up test, and push-up test between T1 and T3 (*P*<.001-.04). For the CG, significant differences were observed in body mass, height, BMI, corrected girth, hips girth, waist to hip ratio, and muscle mass between T1 and T2 (*P*<.001-.02); in corrected girths, hips girth, muscle mass, and CMJ test between T2 and T3 (*P*<.001-.03); and in height, corrected girths, hips girth, waist to hip ratio, muscle mass, CMJ test, and curl-up test between T1 and T3 (*P*<.001-.01).

The effect of the covariate gender on the study variables is shown in Multimedia Appendix 2. It was a determinant factor in the differences found in the EG in PA level, body mass, height, sum of 3 skinfolds, corrected girths, hips girth, waist to hip ratio, muscle mass, VO_2max_, CMJ test, curl-up test, and push-up test between T1 and T2 (*P*<.001-.04); in PA level, sum of 3 skinfolds, corrected arm girth, and VO_2max_ between T2 and T3 (*P*<.001-.04); and in height, corrected girths, hips girth, waist to hip ratio, muscle mass, CMJ test, curl-up test, and push-up test between T1 and T3 (*P*<.001-.01). In the CG, this covariate was a determinant factor in the differences found in body mass, height, BMI, corrected girths, hips girth, waist to hip ratio, and muscle mass between T1 and T2 (*P*<.001-.047); in BMI, corrected girths, hips girth, and muscle mass between T2 and T3 (*P*<.001-.002); and in height, corrected girths, hips girth, waist to hip ratio, muscle mass, CMJ test, and curl-up test between T1 and T3 (*P*<.001-.02).

The covariate specific app used (Multimedia Appendix 3) was not shown to be influential either in the differences found in PA level or in anthropometry variables and body composition, although it was influential in the changes in VO_2max_ (T1-T2: *P*=.004; T2-T3: *P*=.007) and the curl-up test (T1-T2: *P*<.001; T1-T3: *P*<.001).

### Differences Between the EG and CG in the Study Variables at the Same Time Point During the Research Period

Table 3 shows the differences between the EG and CG in the study variables at the 3 time points (T1, T2, and T3). The differences at the 3 time points were significant in the sum of 3 skinfolds (*P*=.01-.03), in hips girth (*P*=.003-.02), and fat mass (*P*=.02-.03), as well as in the curl-up test at T2 (*P*=.047). The rest of the variables showed no significant differences between the 2 groups at any of the time points.

**Table 3:** Differences between the experimental group (EG) and control group (CG) at the study time points (intergroup differences).

Variable and time point			EG, mean (SD)		CG, mean (SD)		Mean difference (EG–CG)		*P* value		*F* test (*df*)		η_p_²
**Subjective level of physical activity**													
	T1	2.62 (0.68)		2.72 (0.64)		–0.100		.20		1.664 (1)		0.005	
	T2	2.79 (0.59)		2.72 (0.73)		0.074		.29		1.104 (1)		0.003	
	T3	2.68 (0.68)		2.66 (0.71)		0.024		.75		0.749 (1)		0.001	
**Body mass (kg)**													
	T1	55.16 (12.87)		52.56 (10.84)		2.594		.05		3.812 (1)		0.011	
	T2	56.06 (12.69)		53.51 (10.71)		2.549		.05		3.778 (1)		0.011	
	T3	56.03 (11.63)		53.71 (10.72)		2.319		.06		2.805 (1)		0.010	
**Height (cm)**													
	T1	162.35 (9.04)		161.02 (8.82)		1.335		.17		1.852 (1)		0.005	
	T2	163.11 (8.98)		161.63 (8.77)		1.482		.13		2.313 (1)		0.007	
	T3	163.27 (9.74)		162.25 (9.31)		1.021		.33		0.949 (1)		0.003	
**BMI (kg/m^2^)**													
	T1	20.87 (3.84)		20.19 (3.34)		0.682		.09		2.896 (1)		0.008	
	T2	20.98 (3.69)		20.44 (3.21)		0.538		.16		1.956 (1)		0.006	
	T3	20.93 (3.69)		20.26 (3.19)		0.662		.09		2.972 (1)		0.009	
**Sitting height (cm)**													
	T1	84.75 (9.59)		82.85 (12.28)		1.893		.10		2.661 (1)		0.007	
	T2	85.54 (4.78)		83.43 (11.18)		2.112		.07		6.001 (1)		0.017	
	T3	82.90 (19.43)		83.12 (15.32)		–0.215		.91		0.012 (1)		0.001	
**Sum of 3 skinfolds (mm)**													
	T1	52.03 (26.58)		45.05 (24.18)		6.979		.01		6.111 (1)		0.018	
	T2	50.35 (24.51)		44.44 (23.30)		5.908		.03		4.989 (1)		0.014	
	T3	51.50 (25.40)		45.12 (23.80)		6.377		.02		5.472 (1)		0.016	
**Corrected arm girth (cm)**													
	T1	20.83 (2.77)		20.81 (2.75)		0.012		.97		0.002 (1)		0.001	
	T2	21.26 (2.79)		21.20 (2.67)		0.055		.85		0.034 (1)		0.001	
	T3	21.48 (2.83)		21.49 (2.67)		–0.013		.97		0.002 (1)		0.001	
**Corrected thigh girth (cm)**													
	T1	39.18 (4.78)		39.43 (5.24)		–0.252		.65		0.212 (1)		0.001	
	T2	40.11 (4.57)		39.89 (4.22)		0.218		.66		0.200 (1)		0.001	
	T3	40.19 (4.73)		40.64 (4.40)		–0.443		.38		0.765 (1)		0.002	
**Corrected calf girth (cm)**													
	T1	28.95 (3.55)		28.75 (2.75)		0.200		.58		0.312 (1)		0.001	
	T2	29.27 (2.91)		29.28 (2.66)		–0.009		.98		0.001 (1)		0.001	
	T3	29.35 (2.90)		29.37 (2.68)		–0.021		.95		0.005 (1)		0.001	
**Waist girth (cm)**													
	T1	68.36 (8.84)		67.57 (7.13)		0.792		.38		0.769 (1)		0.002	
	T2	68.44 (8.44)		67.84 (7.24)		0.599		.50		0.464 (1)		0.001	
	T3	68.48 (9.05)		68.01 (7.49)		0.470		.61		0.255 (1)		0.001	
**Hips girth (cm)**													
	T1	89.24 (9.25)		86.40 (7.88)		2.840		.003		8.745 (1)		0.025	
	T2	90.18 (8.74)		87.67 (7.83)		2.507		.007		7.380 (1)		0.021	
	T3	90.55 (8.77)		88.31 (7.96)		2.248		.02		5.835 (1)		0.017	
**Waist to hip ratio**													
	T1	0.77 (0.05)		0.78 (0.05)		–0.017		.002		9.392 (1)		0.027	
	T2	0.76 (0.05)		0.77 (0.05)		–0.016		.005		7.818 (1)		0.022	
	T3	0.76 (0.06)		0.77 (0.06)		–0.015		.02		5.808 (1)		0.017	
**Muscle mass (kg)**													
	T1	17.91 (5.05)		18.38 (4.74)		–0.473		.38		0.759 (1)		0.002	
	T2	18.60 (5.11)		18.85 (4.48)		–0.247		.65		0.213 (1)		0.001	
	T3	18.85 (5.18)		19.39 (4.65)		–0.540		.33		0.970 (1)		0.003	
**Fat mass (%)**													
	T1	22.73 (10.23)		20.10 (10.12)		2.630		.02		5.505 (1)		0.016	
	T2	22.20 (9.84)		19.79 (9.90)		2.414		.03		4.947 (1)		0.014	
	T3	22.31 (9.90)		19.89 (9.75)		2.415		.03		4.973 (1)		0.014	
**VO_2max_^a^ (ml/kg/min)**													
	T1	38.03 (4.89)		38.76 (5.10)		–0.734		.20		1.620 (1)		0.005	
	T2	39.03 (5.71)		39.39 (5.10)		–0.359		.57		0.320 (1)		0.001	
	T3	38.12 (6.69)		39.16 (6.49)		–1.044		.17		1.855 (1)		0.006	
**CMJ^b^ test (cm)**													
	T1	21.82 (7.53)		22.40 (7.01)		–0.576		.47		0.529 (1)		0.001	
	T2	23.16 (7.93)		22.97 (9.26)		0.189		.84		0.043 (1)		0.001	
	T3	23.19 (8.16)		24.56 (8.60)		–1.368		.13		2.306 (1)		0.006	
**Curl-up test (repetitions, n)**													
	T1	20.51 (11.49)		20.99 (11.10)		–0.478		.70		0.146 (1)		0.001	
	T2	24.31 (10.69)		22.53 (12.26)		1.773		.047		2.011 (1)		0.006	
	T3	24.80 (11.28)		24.07 (11.71)		0.730		.56		0.334 (1)		0.001	
**Push-up test (repetitions, n)**													
	T1	6.80 (9.41)		7.64 (9.24)		–0.844		.44		0.597 (1)		0.002	
	T2	8.72 (10.95)		8.62 (9.66)		0.101		.93		0.007 (1)		0.001	
	T3	7.93 (10.45)		8.36 (10.27)		–0.433		.72		0.127 (1)		0.001	

^a^VO_2max_: maximal oxygen uptake.

^b^CMJ: countermovement jump.

Regarding the intergroup differences, it is striking that the differences found in the sum of 3 skinfolds, hips girth, and fat mass were not influenced by the covariate maturity status. In the case of the covariate gender, it could be a determinant factor in the differences found at T1 in PA level, body mass, height, sitting height, corrected calf girth, hips girth, and muscle mass (*P*=.003-.04); at T2 in body mass, height, sitting height, corrected girths, hips girth, muscle mass, VO_2max_, CMJ test, curl-up test, and push-up test (*P*<.001-.048); and at T3 in body mass, height, corrected calf girth, and muscle mass (*P*=.01-.03). Finally, the covariate specific app used seemed to influence the differences found in the sum of 3 skinfolds, hips girth, waist to hip ratio, and fat mass at T1, T2, and T3 (*P*=.002-.04), in the curl-up test at T2 (*P*=.04) and T3 (*P*=.02), and in VO_2max_ at T3 (*P*=.02; Multimedia Appendix 4).

Table 4 shows the differences in the changes produced between the EG and CG when comparing the different time points (T1–T2, T1–T3, and T2–T3). The results showed that the changes produced in PA level (*P*=.004) and the curl-up test (*P*=.02) were significantly higher in the EG than in the CG between T1 and T2. In addition, the changes in corrected thigh girth (*P*=.003) and muscle mass (*P*=.02) between T2 and T3 were greater in the CG than in the EG. In the rest of the variables, the changes between the EG and the CG at the different time points were not significant.

**Table 4:** Differences in the changes produced between the experimental group (EG) and control group (CG) when comparing T1–T2, T1–T3, and T2–T3.

Variable	T1: EG–CG	T2: EG–CG	T3: EG–CG	Mean difference (T1–T2)	*P* value	Mean difference (T1–T3)	*P* value	Mean difference (T2–T3)	*P* value
Subjective level of physical activity	–0.092	0.074	0.024	–0.166	.004	–0.116	.08	0.050	.46
Body mass (kg)	2.594	2.549	2.319	0.052	.80	0.273	.52	0.221	.57
Height (cm)	1.335	1.482	1.021	–0.142	.45	0.317	.51	0.459	.31
BMI (kg/m^2^)	0.682	0.538	0.662	0.146	.06	0.018	.84	–0.128	.06
Sitting height (cm)	1.893	2.112	–0.215	–0.278	.83	1.114	.20	2.278	.08
Sum of 3 skinfolds (mm)	6.979	5.908	6.377	1.072	.28	0.602	.57	–0.470	.50
Corrected arm girth (cm)	0.012	0.055	–0.013	–0.043	.62	0.025	.81	0.068	.41
Corrected thigh girth (cm)	–0.252	0.218	–0.443	–0.470	.07	0.191	.53	0.661	.003
Corrected calf girth (cm)	0.200	–0.009	–0.021	0.209	.33	0.221	.32	0.012	.89
Waist girth (cm)	0.792	0.599	0.470	0.193	.43	0.322	.32	0.129	.60
Hips girth (cm)	2.840	2.507	2.248	0.333	.20	0.593	.07	0.259	.26
Waist to hip ratio	–0.017	–0.016	–0.015	–0.001	.52	–0.002	.58	–0.001	.84
Muscle mass (kg)	–0.473	–0.247	–0.540	–0.226	.12	0.067	.71	0.292	.02
Fat mass (%)	2.630	2.414	2.415	0.216	.56	0.215	.58	–0.001	.99
VO_2max_^a^ (ml/kg/min)	–0.734	–0.359	–1.044	–0.374	.28	0.311	.53	0.685	.14
CMJ^b^ test (cm)	–0.576	0.189	–1.368	–0.765	.37	0.792	.34	1.557	.06
Curl-up test (repetitions, n)	–0.478	1.773	0.730	–2.747	.02	–1.242	.26	1.505	.18
Push-up test (repetitions, n)	–0.844	0.101	–0.433	–0.685	.33	–1.000	.17	–0.314	.71

^a^VO_2max_: maximal oxygen uptake.

^b^CMJ: countermovement jump.

Regarding the influence of the covariates on the changes found between the EG and CG at the different time points (Multimedia Appendix 5), it was observed that none of the covariates had any influence either on the changes found between T1 and T2 in PA level or on the changes in the muscle mass between T2 and T3. However, changes in the curl-up test were influenced by the covariate gender between T1 and T2 (*P*=.04), just as the changes in the corrected thigh girth were influenced by gender (*P*=.04) and specific app used (*P*=.01) between T2 and T3. The covariate maturity did not have a significant influence on any of the changes (*P*=.09-.97).

## Discussion

### Summary of the Main Results of the Study

The results of this research show that after the use of the step tracker mobile apps became voluntary and was no longer promoted as a PE class assignment, only a small percentage of adolescents (18/216, 8.3%) continued using them for PA, with minimal training volume. Comparing the changes in the study variables in the EG and CG (intragroup differences) before and after the mandatory and promoted period (T1 vs T2), the EG showed an increase in PA level and fitness variables, with a decrease in the sum of 3 skinfolds. However, at the end of the nonmandatory and nonpromoted period, there was a decrease in adolescents’ PA level and VO_2max_, accompanied by an increase in the sum of 3 skinfolds, compared to the values at the end of the mandatory and promoted period (T2 vs T3). Both EG and CG exhibited increases in corrected arm girth, hips girth, and muscle mass. Finally, when comparing the measurements taken before the start of the mandatory and promoted period and at the end of the nonmandatory and nonpromoted period (T1 vs T3), both groups showed significant increases in body mass, height, corrected girths, hips girth, waist to hip ratio, muscle mass, CMJ test, and curl-up test, as well as an increase in the push-up test only in the EG. These changes were influenced by the covariates maturity status and gender (both of which influenced most of the variables related to PA level, anthropometric measurements, body composition, and fitness in both groups) as well as by the covariate specific app used (which mainly influenced VO_2max_ and the curl-up test).

Regarding intergroup differences, significant differences were noted between the EG and CG at T1, T2, and T3 in sum of 3 skinfolds, hips girth, waist to hip ratio, and fat mass, and at T2 in the curl-up test. While maturity status did not affect intergroup differences, gender and specific app used did have an influence. Despite the intra- and intergroup differences found during both intervention periods, greater changes from T1 to T2 were seen in the EG, particularly in PA level and the curl-up test, unaffected by the covariates, except for gender in the curl-up test. Conversely, from T2 to T3, the CG exhibited greater changes in corrected thigh girth, being influenced by gender and specific app used, and muscle mass, with no covariate influence. No significant changes were found between T1 and T3, with no influence of the covariates.

### Purpose of This Study

The study addresses the challenge of promoting PA among adolescents due to limited school hours and motivation issues [50], which makes it impossible to meet the World Health Organization recommendations [48]. Previous research suggested that mandatory app use promoted as a PE class assignment could enhance PA, body composition, and fitness during the first week of use due to the apps’ novelty [9,51]. However, it is unclear whether these effects persist when app use becomes nonmandatory and nonpromoted. For this reason, the study aims to assess whether step tracker mobile apps could encourage PA outside of school hours and establish walking as a healthy habit.

### Use of Apps by Adolescents During Mandatory and Promoted and Nonmandatory and Nonpromoted Use Periods

As the results show, during the nonmandatory and nonpromoted intervention period, only a small percentage of adolescents (18/216, 8.3%) used the mobile apps; therefore, the changes achieved during the mandatory and promoted intervention period faded away. These results are similar to those of the study by Slootmaker et al [52], in which the use of wearable devices and websites led to improvements in PA after 3 months of the intervention, although the effects disappeared after 8 months when participants did not use the devices for 5 months. One possible explanation for these results is that PA during adolescence is strongly influenced by intrinsic motivation [53,54] and enjoyment experienced during PA [55]. Knowing this, it is possible that walking with an electronic device is not the most satisfying activity for adolescents; therefore, once the extrinsic component, such as the incentive of a bonus point in the PE grade, is removed, all interest shown initially in the intervention is lost. Thus, if the intention is for this population to use these apps due to the benefits they provide on PA level, body composition, and fitness [9,18,43], their use can be made mandatory and promoted by the school, at least as far as walking for exercise is concerned.

These findings suggest that interventions using step tracker mobile apps, when mandatory and promoted as a PE class assignment, may not establish an independent walking habit among adolescents. The primary goal should be to promote lasting habits because short-term increases in PA, while beneficial for fitness and body composition, lack long-term impact. Perhaps 1 of the main drawbacks that prevents adolescents from adhering to the use of these apps is their mandatory implementation by educational institutions; when their use is not promoted or rewarded, they cease to be effective [56]. This indicates that students are participating in the intervention for the reward (ego orientation), rather than for the benefits it might have on their present and future health (task orientation). Furthermore, it would be necessary to consider whether this type of intervention is less effective because it focuses only on cardiorespiratory improvement, and whether the inclusion of other types of training, such as strength or flexibility, would increase adherence [56] because they are more novel or closer to adolescents’ interests.

Considering the results obtained in this study, the first research hypothesis (H1), which proposed that adolescents will stop using the step tracker mobile apps during the period of nonmandatory and nonpromoted use, can be accepted. During the period of nonpromoted and nonmandatory use, only a few of the adolescents (18/216, 8.3%) continued to use the apps, and the distance walked was minimal. This suggests that the return to baseline levels after the period of nonmandatory and nonpromoted use is due to the fact that the adolescents did not continue walking for exercise, which is why the increase in energy expenditure and improvement in physical fitness achieved during the period of mandatory and promoted use was lost during this period.

### Effects of Interventions on PA Level Among Adolescents

The EG demonstrated an increase in PA level during the mandatory and promoted intervention, surpassing the CG. However, these benefits were not sustained over time. The findings align with previous research, which demonstrated that the use of mobile apps led to a notable rise in adolescents’ PA level [9,17]. Notably, this study adds a new perspective, indicating that the effectiveness of step tracker mobile apps diminishes once their use is nonmandatory and nonpromoted as a PE class assignment. This highlights the importance of enforcing app use for enhancing adolescent PA. Future interventions should consider this because effectiveness may hinge on mandatory use. Future research is needed to promote the nonmandatory use of step tracker mobile apps in adolescents who have not previously used such apps because this would allow us to demonstrate whether it is the lack of mandatory use or the loss of interest in the use of the apps that leads to nonuse.

### Effects of Interventions on the Kinanthropometric and Body Composition Variables Among Adolescents

Regarding the kinanthropometric and body composition variables, height and body mass increased significantly in all groups throughout the study, consistent with typical growth patterns during puberty [57]. During PHV, which typically occurs between the age of 11.4 and 12.2 years in female individuals and 13.8 and 14.4 years in male individuals, the height of female individuals and male individuals increases steadily [58,59]. Regarding body mass, previous research has shown similar results [9], and 1 possible explanation for this finding is that the body mass variable does not allow discriminating whether the change produced was due to an improvement in muscle mass or fat mass [60]. In this study, corrected girths and muscle mass also increased significantly in both groups, suggesting potential impacts of maturation rather than app-specific exercises. If the apps used had included strength exercises, it could be speculated that these exercises were the cause of the improvements in girths and muscle mass, as observed in previous research [61]. However, the exclusive use of apps meant for aerobic training makes us consider that the changes were the consequence of the maturation process of these adolescents, characterized by hormonal changes related to increases in muscle mass [62,63]. These results are corroborated with the inclusion of the covariates maturity status and gender. The maturational state had an influence on the intragroup differences in muscle mass variables but did not influence the intergroup differences at the different time points (EG vs CG at T1, T2, or T3), which establishes the importance of the maturational state in the changes found and also agrees with previous research, which showed that limb girths increase during the maturational process [64]. In addition, it should be noted that the covariate gender also influenced the changes in muscle mass during the different time points, which could be due to the fact that during puberty, muscle development is greater in male individuals than in female individuals due to the higher production of steroid hormones in male individuals, with clear differences between both genders [62].

With respect to the fat variables, the EG experienced a significant decrease in the sum of 3 skinfolds between T1 and T2, but this change reverted to baseline levels at T3. This aligns with previous findings suggesting that the use of step tracker mobile apps can reduce fat mass in adolescents [9]. Increased PA during the mandatory and promoted period likely contributed to this reduction, increasing energy expenditure, as seen in other 10-week aerobic exercise programs in the adolescent population [65], which could have influenced the decrease in fat mass. The main novelty of this study is that when app promotion ceased, fat mass returned to initial levels, echoing findings of detraining studies [20]. This could be because the increase in PA achieved during the period of mandatory and promoted use of the app was lost when the use became nonmandatory and nonpromoted, which could have prevented the adolescents from maintaining their increased energy expenditure. In addition, maturity status and gender did not impact fat mass changes, suggesting the intervention’s influence. Nevertheless, the small effect sizes and nonsignificant differences between the groups hint at inconsistency in app-induced changes, possibly explaining the return to baseline values after the intervention. Therefore, future research with the use of step tracker mobile apps for a longer period, which also considers other relevant variables for aerobic training to be effective, such as duration or intensity [66,67], is needed to elucidate their true effect on adolescent body composition.

In hips girth, a significant increase was found in both groups between T1 and T2, as well as T1 and T3. A possible explanation for these results is that hips girth steadily increases during adolescence, ending with a plateau at age 16 years [68], which could explain the increase in this variable in the 2 groups. It would be important for future research to analyze these differences according to the age or ethnicity of the adolescents because these variables significantly influence hips girth [69].

### Effects of Interventions on the Physical Fitness Variables Among Adolescents

As for the physical fitness tests, a significant increase in VO_2max_ was only found in the EG between T1 and T2, with a significant decrease observed between T2 and T3. These results are similar to previous research, in which VO_2max_ increased and performance in the 20-meter shuttle run test improved after the period of mobile app use compared to the CG [9,10]. A possible explanation for these results could be that the use of the apps during the mandatory and promoted period, in which an incentive was offered, favored the improvement in the adolescents’ physical fitness. However, when its use became nonmandatory and nonpromoted as a PE class assignment, and it was no longer incentivized, it is possible that the adolescents did not walk a sufficient distance or at the intensity necessary to maintain the significant improvements achieved in physical fitness, with these variables significantly influencing VO_2max_ [70]. However, future research analyzing the intensity and volume of adolescents’ walks while using step tracker mobile apps is needed to provide more information in this area and to discover whether the maintenance of the benefits obtained is also dependent on these factors.

In the CMJ, curl-up, and push-up tests, it was observed that in the EG, performance improved between T1 and T2 and remained high at T3, while in the CG, the adolescents showed an improvement at T3 compared to T1, with no differences found in the push-up test. These results are similar to previous research in which improvements in the curl-up and push-up tests were found after the mandatory and promoted period of use of mobile apps [9], as well as in specific 6-week aerobic walking programs [71]. Walking has been shown to improve trunk muscle strength [72], which, together with the increases found in this study in muscle mass and corrected girths, could be the reason why the EG showed significant improvements in these fitness tests. However, maturity status, gender, and specific app used should be considered when analyzing changes in physical fitness because they seem to be relevant in the differences found, and future research is needed to learn about the differences in the benefits obtained with the mobile apps as a function of these factors.

In addition, the improvements in the curl-up test in the CG exclusively occurred at T3 compared to T1; this may be solely due to changes in muscle mass caused by the maturation process [62,63], which would be slower in the adolescents in the CG than in the adolescents in the EG who used the app and would favor the improvement in trunk musculature, explaining the absence of differences between T1 and T2 and between T2 and T3 in this group. This was observed with the inclusion of the covariate maturity status because it was shown to influence the differences in the curl-up test of the adolescents in the CG between T1 and T3. In addition, it should be noted that the changes between T1 and T2, when comparing the EG and CG, were significantly greater in the EG, which could be consistent with the fact that walking improves trunk muscle strength [72] because the covariate maturity status did not influence the differences in the changes between the 2 groups. Therefore, the use of step tracker mobile apps could be of interest for improving performance in fitness tests that require trunk strength, allowing for improvements that are superior to those achieved solely due to the maturation process.

One of the unexpected aspects of this study is that the covariate specific app used influenced the results obtained on the adolescents’ body composition and fitness. In 1 of the first studies on the subject, it was found that the step tracker specific app used had almost no influence on the benefits obtained with a compulsory PE class intervention [9]. However, this study has shown that the covariate specific app used can be a determining factor in the changes obtained; therefore, future research should analyze the causes of these differences to try to find the reasons why they occur and whether this could depend on whether adolescents feel more comfortable with a particular app and prefer using this app over another, which would open the door to research that could delve deeper into the specific components included in each app and how they are valued by adolescents.

The second research hypothesis (H2), which proposed that there will be significant differences in adolescents’ PA level, body composition, and physical fitness during the mandatory and promoted period, influenced by maturity status and gender but not by specific app used, can be partially accepted. This is because the results showed benefits in the PA level, cardiorespiratory fitness, and fat variables after the mandatory and promoted intervention, although the only significant changes observed between the EG and CG were in abdominal strength and PA level between T1 and T2. Furthermore, as expected, the covariates maturity status and gender influenced the results observed in body composition and fitness, although specific app used also had an influence.

The third research hypothesis (H3), which stated that some of the benefits achieved by the adolescents during the mandatory and promoted period will be lost after the nonmandatory and nonpromoted period, with the results being influenced by maturity status and gender but not by specific app used, can be partially accepted. The benefits obtained during the mandatory and promoted period were lost when adolescents stopped using the apps during the nonmandatory and nonpromoted period. Only the benefits in muscle mass and fitness variables (CMJ, curl-up, and push-up tests) were maintained at T3 compared to T1 in the EG. However, the changes between T1 and T3, when comparing the EG and CG, were not significant in any of the variables analyzed. Again, the covariates maturity status, gender, and specific app used influenced the results.

### Limitations of This Study

This study is not without limitations. First, the measurement of PA using a questionnaire has limitations; for example, although some studies show that the PAQ-A can be valid for measuring changes in PA performed by the same group in 2 different time periods [30], others indicate that its validity and reliability are questionable because it does not measure aspects such as frequency or intensity of activity, and nor does it allow for comparisons of changes in PA between 2 groups [73]. Therefore, although the PAQ-A is sometimes the best choice for measuring PA due to its favorable cost-benefit ratio [74,75], its use could affect the results of the study. Future research should include accelerometry, which would also make it possible to differentiate the intensities and the time spent in each activity level, which would make it possible to analyze whether these factors are modulators of change. Second, in future research, it would be appropriate to consider aspects such as the volume and intensity of training performed with step tracker mobile apps because these aspects can influence changes in body composition and fitness, as well as the length of time for which the changes achieved are maintained [76,77]. Third, the nutritional intake of adolescents was not considered in this study, and although previous research has shown that aspects of nutrition, such as adherence to the Mediterranean diet, are not modified with the use of step tracker mobile apps [9], it would be important to consider the amount and type of nutritional intake because these variables can influence changes in body composition [78]. Fourth, only those students who completed at least 25% of the training volume were included in the analyses. This is because previous research has shown that this is the minimum volume needed for differences in adolescent body composition and fitness to begin to occur [43]. In this study, when trying to analyze whether adolescents continued to use the app when it was neither mandatory nor promoted, it was assumed that those who did not use the apps during the mandatory and promoted period would not use them in the nonmandatory and nonpromoted period either. Therefore, this may be a bias because adolescents who did not even start the intervention were eliminated from the analysis, but this was not the aim of the research. Finally, another noteworthy aspect to be considered could be that this type of intervention does not consider the context or the environment of the adolescents; while it places the adolescent at the center of the intervention, it does not consider that other agents in their close environment (family and friends) could be of great relevance for the acquisition of healthy habits [79,80]. Furthermore, another unexplored aspect that should be considered is that at these ages (12-16 y), adolescents practice physical sports activities mainly for their competitive and recreational component [53-55], not for their health benefits, and walking may not be the most motivating and fun activity for this population, which may hinder the establishment of this healthy habit when the performance of these tasks is optional for students, although improvements in health are evident.

### Practical Applications

Considering the limitations of this research, a practical application derived from it is that if step tracker mobile apps are intended to be used to increase daily steps and PA level of the adolescent population and are promoted as a PE class assignment, their use must be maintained over time or accompanied by other types of complementary programs that promote the establishment of healthy lifestyle habits [81] that allow the improvements obtained to be maintained. In this regard, previous research has shown that the use of step tracker mobile apps, combined with nutritional programs or training sessions on healthy habits, has a significant effect on improving the health status of adolescents by facilitating their continued use [81,82]. On the contrary, the occasional use of these apps is ineffective; once the period of mandatory and promoted use ends, adolescents who used the apps will return to baseline levels that are similar to those of adolescents who did not use them as a result of detraining, as observed in previous PA promotion programs where young people who undertook aerobic training showed losses in the benefits obtained after a period of detraining [20].

### Conclusions

This study is the first to analyze the losses that occur in the positive changes achieved in PA level, body composition, and fitness variables by adolescents aged 12 to 16 years after a period of mandatory and promoted use of step tracker mobile apps when their use becomes nonmandatory and nonpromoted. Our findings suggest that adolescents stop using step tracker mobile apps when their use is neither mandatory nor promoted as a PE class assignment. During the mandatory and promoted period, adolescents in the EG increased their PA level and cardiorespiratory fitness and reduced their fat mass. However, when participation in the step increase program with the apps was neither mandatory nor promoted, adolescents stopped using the apps and discontinued their walking practice; as a consequence, the gains achieved were lost, leading to a regression to baseline levels. The change in the EG compared to the CG was only significant in the curl-up test at the end of the mandatory and promoted intervention but not in the rest of the variables or in the comparison between the other time points. Therefore, this study shows that the use of mobile apps by adolescents aged 12 to 16 years in their free time, promoted as a PE class assignment, did not manage to create a healthy walking habit in this population, which could be a determining factor in fostering independent walking practice in adolescents, which could yield significant health benefits. These results are of relevance for the use of step tracker mobile apps in education because when their use is mandated and promoted as a PE class assignment, they seem effective in reducing fat mass and increasing PA level in adolescents. Special attention should be paid to the covariates maturity status, gender, and specific app used because they might influence the changes achieved during the intervention. Future research along these lines should also analyze the most influential aspects to be considered to achieve independent use and greater adherence of adolescents to step tracker mobile apps, including analyses of their immediate environment and the factors that could be most relevant, to ensure that use is continued after they have already become familiar with the step tracker mobile app, and its use is neither mandatory nor promoted as a PE class assignment.

## Appendix

Multimedia Appendix 1 Effect of the covariate maturity status in the intragroup (T1 vs T2; T1 vs T2 and T2 vs T3) differences.

Multimedia Appendix 2 Effect of the covariate gender in the intragroup (T1 vs T2; T1 vs T2 and T2 vs T3) differences.

Multimedia Appendix 3 Effect of the covariate specific app used in the intragroup (T1 vs T2; T1 vs T2 and T2 vs T3) differences in the experimental group.

Multimedia Appendix 4 Effect of the covariates maturity status, gender, and app used in the intergroup (experimental group vs control group) differences.

Multimedia Appendix 5 Effect of the covariates maturity status, gender, and app used in the differences in the changes produced between the experimental and control groups.

Multimedia Appendix 6 CONSORT e-HEALTH checklist (V 1.6.1).

## Abbreviations

CG: control group
CMJ: countermovement jump
CONSORT: Consolidated Standards of Reporting Trials
EG: experimental group
PA: physical activity
PAQ-A: Physical Activity Questionnaire for Adolescents
PE: physical education
PHV: peak height velocity
VO2max: maximal oxygen uptake
