# Supplementary material for: Physical Activity, Body Composition, and Fitness Variables in Adolescents After Periods of Mandatory, Promoted or Nonmandatory, Nonpromoted Use of Step Tracker Mobile Apps: Randomized Controlled Trial
Source: JMIR Mhealth Uhealth. 2024 Jul 30;12:e51206. doi: 10.2196/51206 (PMC11322691; doi:10.2196/51206)
Supplement: Multimedia Appendix 1 [file mhealth_v12i1e51206_app1.docx]

Supplementary Table 1. Effect of the covariate maturity status in the intra-group (T1 Vs T2; T1 Vs T2 and T2 Vs T3) differences.

| Variable | Group | T1-T2 | | T1-T3 | | T2-T3 | | F | η2 |
| --- | --- | --- | --- | --- | --- | --- | --- | --- | --- |
|  |  | App use*Maturity | | App use*Maturity | | App use*Maturity | |  |  |
|  |  | Mean Diff | *P* | Mean Diff | *P* | Mean Diff | *P* |  |  |
| Physical Activity Level | Intervention | -0.166 | <.001 | -0.047 | .81 | 0.119 | .02 | 10.405 | 0.056 |
|  | Control | -0.007 | 1.00 | 0.045 | 1.00 | 0.052 | .99 | 0.525 | 0.003 |
| Body mass (kg) | Intervention | -0.970 | <.001 | -0.858 | .13 | 0.111 | 1.00 | 28.701 | 0.144 |
|  | Control | -0.842 | <.001 | -0.830 | .35 | 0.012 | 1.00 | 14.185 | 0.077 |
| Height (cm) | Intervention | -0.850 | <.001 | -1.020 | .003 | -0.171 | 1.00 | 26.750 | 0.135 |
|  | Control | -0.479 | .003 | -1.072 | .01 | -0.593 | .28 | 7.252 | 0.041 |
| BMI (kg/m^2^) | Intervention | -0.120 | .05 | -0.061 | .85 | 0.059 | .47 | 3.245 | 0.019 |
|  | Control | -0.230 | <.001 | -0.056 | 1.00 | 0.174 | .002 | 10.277 | 0.057 |
| Sitting height (cm) | Intervention | -0.440 | 1.00 | 1.373 | .69 | 1.813 | .41 | 1.181 | 0.007 |
|  | Control | 0.653 | .86 | 1.380 | .98 | 0.728 | 1.00 | 0.990 | 0.006 |
| Sum of 3 skinfolds (mm) | Intervention | 1.796 | .01 | 0.604 | 1.00 | -1.192 | .03 | 6.043 | 0.034 |
|  | Control | 0.424 | 1.00 | -0.177 | 1.00 | -0.601 | .86 | 0.612 | 0.004 |
| Corrected arm girth (cm) | Intervention | -0.433 | <.001 | -0.669 | <.001 | -0.236 | <.001 | 53.513 | 0.239 |
|  | Control | -0.382 | <.001 | -0.646 | <.001 | -0.264 | <.001 | 32.003 | 0.158 |
| Corrected thigh girth (cm) | Intervention | -0.913 | <.001 | -1.022 | <.001 | -0.109 | 1.00 | 17.124 | 0.092 |
|  | Control | -0.467 | .07 | -1.182 | <.001 | -0.715 | <.001 | 13.236 | 0.072 |
| Corrected calf girth (cm) | Intervention | -0.352 | .03 | -0.429 | .008 | -0.078 | .48 | 4.708 | 0.027 |
|  | Control | -0.479 | .02 | -0.570 | .004 | -0.090 | .56 | 5.327 | 0.030 |
| Waist girth (cm) | Intervention | -0.093 | 1.00 | -0.106 | 1.00 | -0.012 | 1.00 | 0.189 | 0.001 |
|  | Control | -0.231 | .71 | -0.442 | .25 | -0.211 | .82 | 1.518 | 0.009 |
| Hips girth (cm) | Intervention | -0.951 | <.001 | -1.333 | <.001 | -0.382 | .03 | 21.761 | 0.113 |
|  | Control | -1.236 | <.001 | -1.861 | <.001 | -0.624 | .002 | 26.621 | 0.135 |
| Waist/hip ratio | Intervention | 0.007 | <.001 | 0.010 | <.001 | 0.003 | .28 | 17.146 | 0.092 |
|  | Control | 0.008 | <.001 | 0.011 | <.001 | 0.003 | .69 | 13.925 | 0.076 |
| Muscle mass (kg) | Intervention | -0.697 | <.001 | -0.956 | <.001 | -0.259 | .005 | 37.377 | 0.180 |
|  | Control | -0.464 | <.001 | -0.989 | <.001 | -0.525 | <.001 | 24.893 | 0.128 |
| Fat mass (%) | Intervention | 0.553 | .06 | 0.445 | .23 | -0.108 | 1.00 | 2.683 | 0.016 |
|  | Control | 0.271 | 1.00 | 0.180 | 1.00 | -0.091 | 1.00 | 0.444 | 0.003 |
| VO2 max. | Intervention | -1.019 | <.001 | -0.245 | 1.00 | 0.774 | .02 | 11.672 | 0.071 |
|  | Control | -0.587 | .10 | -0.107 | 1.00 | 0.480 | .56 | 2.566 | 0.017 |
| CMJ (cm) | Intervention | -1.318 | .04 | -1.274 | .04 | 0.044 | 1.00 | 4.031 | 0.023 |
|  | Control | -0.260 | 1.00 | -1.929 | .008 | -1.670 | .03 | 5.425 | 0.030 |
| Curl-up | Intervention | -3.253 | <.001 | -4.165 | <.001 | -0.912 | .49 | 19.347 | 0.103 |
|  | Control | -2.180 | .06 | -3.157 | .001 | -0.977 | .67 | 7.183 | 0.041 |
| Push-up | Intervention | -2.134 | <.001 | -1.126 | .02 | 1.008 | .14 | 11.127 | 0.069 |
|  | Control | -0.718 | .68 | -0.772 | .38 | -0.054 | 1.00 | 1.446 | 0.009 |
