# Supplementary material for: Physical Activity, Body Composition, and Fitness Variables in Adolescents After Periods of Mandatory, Promoted or Nonmandatory, Nonpromoted Use of Step Tracker Mobile Apps: Randomized Controlled Trial
Source: JMIR Mhealth Uhealth. 2024 Jul 30;12:e51206. doi: 10.2196/51206 (PMC11322691; doi:10.2196/51206)
Supplement: Multimedia Appendix 2 [file mhealth_v12i1e51206_app2.docx]

Supplementary Table 2. Effect of the covariate gender in the intra-group (T1 Vs T2; T1 Vs T2 and T2 Vs T3) differences.

| Variable | Group | T1-T2 | | T1-T3 | | T2-T3 | | F | η2 | |
| --- | --- | --- | --- | --- | --- | --- | --- | --- | --- | --- |
|  |  | App use*Gender | | App use*Gender | | App use*Gender | |  |  |  |
|  |  | Mean Diff | *P* | Mean Diff | *P* | Mean Diff | *P* |  |  |  |
| Physical Activity Level | Intervention | -0.162 | <.001 | -0.046 | .81 | 0.116 | .02 | 10.197 | 0.055 |  |
|  | Control | -0.015 | 1.00 | 0.038 | 1.00 | 0.052 | .98 | 0.490 | 0.003 |  |
| Body mass (kg) | Intervention | -0.932 | <.001 | -0.640 | .41 | 0.293 | 1.00 | 26.200 | 0.133 |  |
|  | Control | -0.899 | <.001 | -1.160 | .09 | -0.261 | 1.00 | 16.090 | 0.086 |  |
| Height (cm) | Intervention | -0.822 | <.001 | -0.945 | .006 | -0.123 | 1.00 | 24.321 | 0.124 |  |
|  | Control | -0.520 | .001 | -1.185 | .005 | -0.665 | .18 | 8.437 | 0.047 |  |
| BMI (kg/m^2^) | Intervention | -0.104 | .12 | -0.048 | 1.00 | 0.056 | .53 | 2.533 | 0.015 |  |
|  | Control | -0.255 | <.001 | -0.077 | .83 | 0.178 | .002 | 11.797 | 0.065 |  |
| Sitting height (cm) | Intervention | -0.784 | 1.00 | 1.561 | .62 | 2.344 | .21 | 1.706 | 0.010 |  |
|  | Control | -0.603 | 1.00 | 0.164 | 1.00 | 0.767 | 1.00 | 0.216 | 0.001 |  |
| Sum of 3 skinfolds (mm) | Intervention | 1.751 | .02 | 0.731 | .85 | -1.020 | .04 | 5.205 | 0.030 |  |
|  | Control | 0.498 | 1.00 | -0.377 | 1.00 | -0.875 | .35 | 1.259 | 0.007 |  |
| Corrected arm girth (cm) | Intervention | -0.433 | <.001 | -0.669 | <.001 | -0.236 | <.001 | 54.058 | 0.241 |  |
|  | Control | -0.386 | <.001 | -0.649 | <.001 | -0.263 | <.001 | 32.493 | 0.160 |  |
| Corrected thigh girth (cm) | Intervention | -0.900 | <.001 | -1.011 | <.001 | -0.112 | 1.00 | 16.873 | 0.090 |  |
|  | Control | -0.490 | .047 | -1.198 | <.001 | -0.708 | <.001 | 13.519 | 0.073 |  |
| Corrected calf girth (cm) | Intervention | -0.336 | .043 | -0.430 | .008 | -0.094 | .25 | 5.032 | 0.029 |  |
|  | Control | -0.508 | .008 | -0.574 | .003 | -0.066 | .98 | 5.370 | 0.031 |  |
| Waist girth (cm) | Intervention | -0.084 | 1.00 | -0.141 | 1.00 | -0.057 | 1.00 | 0.245 | 0.001 |  |
|  | Control | -0.273 | .49 | -0.411 | .31 | -0.138 | 1.00 | 1.417 | 0.008 |  |
| Hips girth (cm) | Intervention | -0.913 | <.001 | -1.267 | <.001 | -0.354 | .05 | 20.044 | 0.105 |  |
|  | Control | -1.305 | <.001 | -1.977 | <.001 | -0.671 | <.001 | 30.269 | 0.151 |  |
| Waist/hip ratio | Intervention | 0.007 | <.001 | 0.009 | <.001 | 0.003 | .57 | 15.105 | 0.081 |  |
|  | Control | 0.008 | <.001 | 0.013 | <.001 | 0.004 | .25 | 15.926 | 0.085 |  |
| Muscle mass (kg) | Intervention | -0.703 | <.001 | -0.963 | <.001 | -0.260 | .005 | 38.225 | 0.184 |  |
|  | Control | -0.456 | <.001 | -0.978 | <.001 | -0.522 | <.001 | 24.575 | 0.126 |  |
| Fat mass (%) | Intervention | 0.570 | .05 | 0.523 | .11 | -0.047 | 1.00 | 2.951 | 0.017 |  |
|  | Control | 0.253 | 1.00 | 0.060 | 1.00 | -0.193 | .99 | 0.666 | 0.004 |  |
| VO2 max. | Intervention | -1.102 | <.001 | -0.230 | 1.00 | 0.872 | .009 | 14.318 | 0.085 |  |
|  | Control | -0.487 | .21 | -0.196 | 1.00 | 0.291 | 1.00 | 1.689 | 0.011 |  |
| CMJ (cm) | Intervention | -1.451 | .02 | -1.663 | .005 | -0.212 | 1.00 | 5.727 | 0.031 |  |
|  | Control | -0.401 | 1.00 | -1.726 | .02 | -1.325 | .11 | 4.083 | 0.023 |  |
| Curl-up | Intervention | -3.692 | <.001 | -4.334 | <.001 | -0.642 | .98 | 21.646 | 0.113 |  |
|  | Control | -1.690 | .22 | -2.994 | .001 | -1.304 | .32 | 6.603 | 0.037 |  |
| Push-up | Intervention | -1.959 | <.001 | -1.173 | .01 | 0.785 | .36 | 10.138 | 0.063 |  |
|  | Control | -0.914 | .37 | -0.645 | .59 | 0.270 | 1.00 | 1.546 | 0.010 |  |
