# Supplementary material for: Physical Activity, Body Composition, and Fitness Variables in Adolescents After Periods of Mandatory, Promoted or Nonmandatory, Nonpromoted Use of Step Tracker Mobile Apps: Randomized Controlled Trial
Source: JMIR Mhealth Uhealth. 2024 Jul 30;12:e51206. doi: 10.2196/51206 (PMC11322691; doi:10.2196/51206)
Supplement: Multimedia Appendix 3 [file mhealth_v12i1e51206_app3.docx]

Supplementary Table 3. Effect of the covariate app used in the intra-group differences (T1 Vs T2; T1 Vs T2 and T2 Vs T3) in the experimental group.

| Variable | T1-T2 | | T1-T3 | | T2-T3 | | | F | | η2 | |  |
| --- | --- | --- | --- | --- | --- | --- | --- | --- | --- | --- | --- | --- |
|  | App use*App | | App use*App | | App use*App | | |  |  |  |  |  |
|  | Mean Diff | *P* | Mean Diff | *P* | | Mean Diff | *P* | |  | |  | |
| Physical Activity Level | -0.156 | .07 | -0.067 | .67 | | 0.088 | .35 | | 5.375 | | 0.030 | |
| Body mass (kg) | -0.853 | .20 | 0.098 | 1.00 | | 0.952 | .22 | | 14.216 | | 0.077 | |
| Height (cm) | -0.679 | .50 | -0.711 | .41 | | -0.492 | .56 | | 10.514 | | 0.058 | |
| BMI (kg/m^2^) | -0.122 | .18 | 0.010 | 1.00 | | 0.133 | .05 | | 3.970 | | 0.023 | |
| Sitting height (cm) | -0.513 | 1.00 | 2.541 | .35 | | 3.055 | .21 | | 1.681 | | 0.009 | |
| Sum of 3 skinfolds (mm) | 1.793 | .09 | 0.903 | .93 | | -0.891 | .39 | | 2.937 | | 0.017 | |
| Corrected arm girth (cm) | -0.438 | .10 | -0.648 | .08 | | -0.209 | .35 | | 30.411 | | 0.151 | |
| Corrected thigh girth (cm) | -0.963 | .06 | -0.943 | .06 | | 0.020 | 1.00 | | 10.619 | | 0.059 | |
| Corrected calf girth (cm) | -0.364 | .12 | -0.384 | .11 | | -0.021 | 1.00 | | 2.234 | | 0.013 | |
| Waist girth (cm) | 0.073 | 1.00 | 0.547 | .11 | | 0.474 | .06 | | 3.195 | | 0.018 | |
| Hips girth (cm) | -0.887 | .43 | -1.115 | .09 | | -0.228 | .70 | | 10.041 | | 0.056 | |
| Waist/hip ratio | 0.004 | .50 | 0.006 | .31 | | 0.007 | .11 | | 18.789 | | 0.099 | |
| Muscle mass (kg) | -0.700 | .10 | -0.931 | .06 | | -0.232 | .09 | | 21.628 | | 0.113 | |
| Fat mass (%) | 0.579 | .18 | 0.435 | .54 | | -0.143 | 1.00 | | 1.783 | | 0.010 | |
| VO2 max. | -0.927 | .004 | 0.234 | 1.00 | | 1.161 | .007 | | 7.881 | | 0.049 | |
| CMJ (cm) | -1.140 | .32 | -1.553 | .08 | | -0.412 | 1.00 | | 2.617 | | 0.015 | |
| Curl-up | -3.808 | <.001 | -5.018 | <.001 | | -1.210 | .47 | | 16.750 | | 0.090 | |
| Push-up | -1.485 | .08 | -1.367 | .10 | | 0.118 | 1.00 | | 4.823 | | 0.031 | |
