# Supplementary material for: Physical Activity, Body Composition, and Fitness Variables in Adolescents After Periods of Mandatory, Promoted or Nonmandatory, Nonpromoted Use of Step Tracker Mobile Apps: Randomized Controlled Trial
Source: JMIR Mhealth Uhealth. 2024 Jul 30;12:e51206. doi: 10.2196/51206 (PMC11322691; doi:10.2196/51206)
Supplement: Multimedia Appendix 4 [file mhealth_v12i1e51206_app4.docx]

Supplementary Table 4. Effect of the covariates maturity status, gender and app used in the inter-group (experimental group Vs control group) differences.

| Variables | Time point | EG vs CG * Maturity | | | EG vs CG * Gender | | | EG vs CG * App | | |
| --- | --- | --- | --- | --- | --- | --- | --- | --- | --- | --- |
|  |  | Mean Diff. | *P* | η2 | Mean Diff. | *P* | η2 | Mean Diff. | *P* | η2 |
|  |  |  |  |  |  |  |  |  |  |  |
| Physical Activity Level | T1 | -0.019 | .79 | 0.001 | -0.003 | .97 | 0.001 | -0.159 | .19 | 0.005 |
|  | T2 | 0.139 | .05 | 0.011 | 0.145 | .04 | 0.012 | -0.027 | .82 | 0.000 |
|  | T3 | 0.073 | .35 | 0.003 | 0.081 | .29 | 0.003 | -0.022 | .86 | 0.000 |
| Body mass (kg) | T1 | -0.566 | .64 | 0.001 | 3.569 | .008 | 0.020 | 3.547 | .11 | 0.007 |
|  | T2 | -0.439 | .72 | 0.001 | 3.603 | .007 | 0.021 | 3.382 | .13 | 0.007 |
|  | T3 | -0.423 | .71 | 0.001 | 3.049 | .02 | 0.017 | 1.176 | .58 | 0.001 |
| Height (cm) | T1 | -0.867 | .33 | 0.003 | 2.851 | .003 | 0.026 | 2.325 | .16 | 0.006 |
|  | T2 | -0.497 | .59 | 0.001 | 3.154 | <.001 | 0.033 | 2.270 | .17 | 0.006 |
|  | T3 | -0.920 | .36 | 0.002 | 2.612 | .01 | 0.019 | 2.650 | .13 | 0.007 |
| BMI (kg/m^2^) | T1 | 0.042 | .92 | 0.001 | 0.623 | .13 | 0.007 | 0.886 | .19 | 0.005 |
|  | T2 | -0.068 | .86 | 0.001 | 0.472 | .23 | 0.004 | 0.781 | .23 | 0.004 |
|  | T3 | 0.047 | .90 | 0.001 | 0.595 | .13 | 0.007 | 0.712 | .27 | 0.004 |
| Sitting height (cm) | T1 | -0.487 | .29 | 0.003 | 2.398 | .05 | 0.011 | 2.773 | .16 | 0.006 |
|  | T2 | 0.606 | .47 | 0.001 | 2.579 | .00 | 0.024 | 2.280 | .12 | 0.007 |
|  | T3 | -0.480 | .80 | 0.001 | 1.001 | .61 | 0.001 | -1.072 | .74 | 0.000 |
| Sum of 3 skinfolds (mm) | T1 | 4.575 | .11 | 0.007 | 2.570 | .34 | 0.003 | 12.318 | .009 | 0.020 |
|  | T2 | 3.203 | .23 | 0.004 | 1.317 | .60 | 0.001 | 10.958 | .01 | 0.018 |
|  | T3 | 3.794 | .17 | 0.006 | 1.462 | .57 | 0.001 | 10.776 | .02 | 0.016 |
| Corrected arm girth (cm) | T1 | -0.449 | .13 | 0.007 | 0.519 | .07 | 0.009 | -0.136 | .79 | 0.000 |
|  | T2 | -0.398 | .18 | 0.005 | 0.566 | .05 | 0.011 | -0.076 | .88 | 0.000 |
|  | T3 | -0.426 | .16 | 0.006 | 0.539 | .06 | 0.010 | -0.170 | .74 | 0.000 |
| Corrected thigh girth (cm) | T1 | -0.923 | .09 | 0.008 | 0.638 | .22 | 0.004 | -0.461 | .62 | 0.001 |
|  | T2 | -0.477 | .32 | 0.003 | 1.048 | .02 | 0.015 | 0.108 | .90 | 0.000 |
|  | T3 | -0.783 | .10 | 0.014 | 0.451 | .34 | 0.003 | -0.820 | .33 | 0.003 |
| Corrected calf girth (cm) | T1 | -0.228 | .52 | 0.001 | 0.775 | .02 | 0.015 | 0.102 | .87 | 0.000 |
|  | T2 | -0.356 | .25 | 0.004 | 0.603 | .03 | 0.013 | 0.000 | 1.00 | 0.000 |
|  | T3 | -0.369 | .24 | 0.004 | 0.630 | .02 | 0.015 | -0.158 | .76 | 0.000 |
| Waist girth (cm) | T1 | -0.040 | .97 | 0.001 | 1.775 | .06 | 0.011 | 1.668 | .27 | 0.004 |
|  | T2 | -0.178 | .84 | 0.001 | 1.586 | .07 | 0.010 | 1.082 | .46 | 0.002 |
|  | T3 | -0.376 | .69 | 0.001 | 1.504 | .11 | 0.008 | -0.337 | .83 | 0.000 |
| Hips girth (cm) | T1 | 0.424 | .61 | 0.001 | 2.450 | .01 | 6.206 | 4.494 | .005 | 0.022 |
|  | T2 | 0.139 | .86 | 0.001 | 2.058 | .03 | 4.762 | 4.037 | .009 | 0.020 |
|  | T3 | -0.103 | .90 | 0.001 | 1.740 | .07 | 3.360 | 3.402 | .03 | 0.014 |
| Waist/hip ratio | T1 | -0.005 | .35 | 0.003 | -0.002 | .61 | 0.001 | -0.019 | .04 | 0.013 |
|  | T2 | -0.004 | .48 | 0.001 | -0.001 | .86 | 0.000 | -0.021 | .02 | 0.015 |
|  | T3 | -0.004 | .51 | 0.001 | 0.001 | .87 | 0.000 | -0.032 | .002 | 0.027 |
| Muscle mass (kg) | T1 | -1.034 | .06 | 0.010 | 0.957 | .03 | 0.014 | -0.723 | .43 | 0.002 |
|  | T2 | -0.801 | .14 | 0.006 | 1.204 | .005 | 0.023 | -0.483 | .59 | 0.001 |
|  | T3 | -1.068 | .06 | 0.011 | 0.943 | .03 | 0.014 | -0.817 | .37 | 0.002 |
| Fat mass (%) | T1 | 1.749 | .13 | 0.007 | 0.987 | .36 | 0.002 | 4.304 | .02 | 0.015 |
|  | T2 | 1.466 | .18 | 0.005 | 0.670 | .52 | 0.001 | 3.965 | .03 | 0.014 |
|  | T3 | 1.483 | .18 | 0.005 | 0.524 | .61 | 0.001 | 4.062 | .03 | 0.014 |
| VO2 max. | T1 | 0.204 | .71 | 0.001 | 0.393 | .45 | 0.002 | -1.844 | .06 | 0.012 |
|  | T2 | 0.635 | .31 | 0.003 | 1.008 | .05 | 0.011 | -1.670 | .12 | 0.008 |
|  | T3 | 0.342 | .64 | 0.001 | 0.427 | .54 | 0.001 | -2.984 | .02 | 0.017 |
| CMJ (cm) | T1 | -0.312 | .70 | 0.001 | 0.727 | .34 | 0.003 | -2.238 | .09 | 0.008 |
|  | T2 | 0.746 | .43 | 0.002 | 1.777 | .04 | 0.012 | -1.963 | .20 | 0.005 |
|  | T3 | -0.967 | .30 | 0.003 | 0.664 | .41 | 0.002 | -2.576 | .09 | 0.008 |
| Curl-up | T1 | -0.532 | .68 | 0.001 | 1.066 | .39 | 0.002 | 2.104 | .32 | 0.003 |
|  | T2 | 0.540 | .67 | 0.001 | 3.068 | .02 | 0.017 | 4.398 | .04 | 0.013 |
|  | T3 | 0.475 | .72 | 0.001 | 2.406 | .05 | 0.011 | 5.157 | .02 | 0.017 |
| Push-up | T1 | -0.652 | .57 | 0.001 | 1.122 | .26 | 0.004 | -0.994 | .59 | 0.001 |
|  | T2 | 0.764 | .55 | 0.001 | 2.167 | .04 | 0.012 | -1.165 | .58 | 0.001 |
|  | T3 | -0.298 | .81 | 0.001 | 1.651 | .14 | 0.007 | 0.032 | .99 | 0.000 |

EG: Experimental group; CG: Control group.
