# Supplementary material for: Physical Activity, Body Composition, and Fitness Variables in Adolescents After Periods of Mandatory, Promoted or Nonmandatory, Nonpromoted Use of Step Tracker Mobile Apps: Randomized Controlled Trial
Source: JMIR Mhealth Uhealth. 2024 Jul 30;12:e51206. doi: 10.2196/51206 (PMC11322691; doi:10.2196/51206)
Supplement: Multimedia Appendix 5 [file mhealth_v12i1e51206_app5.docx]

Supplementary Table 5. Effect of the covariates maturity status, gender and app used in the differences in the change produced between the experimental and control groups.

| Variable | Descriptors (Mean ± SD) | | | App use*Maturity | | | App use*Gender | | | | | | App use*App | | | | | |
| --- | --- | --- | --- | --- | --- | --- | --- | --- | --- | --- | --- | --- | --- | --- | --- | --- | --- | --- |
|  |  |  |  | T1-T2 | T1-T3 | T2-T3 | T1-T2 | | T1-T3 | | T2-T3 | | T1-T2 | | T1-T3 | | T2-T3 | |
|  | EG-CG T1 | EG-CG T2 | EG-CG T3 | Mean diff. and *P* | Mean diff. and *P* | Mean diff. and *P* | | Mean diff. and *P* | | Mean diff. and *P* | | Mean diff. and *P* | | Mean diff. and *P* | | Mean diff. and *P* | | Mean diff. and *P* |
| Physical Activity Level | -0.092 | 0.074 | 0.024 | -0.099; .10 | -0.025; .70 | 0.121; .06 | | -0.090; .13 | | -0.005; .94 | | 0.085; .21 | | -0.114; .23 | | -0.081; .46 | | 0.033; .76 |
| Body mass (kg) | 2.594 | 2.549 | 2.319 | -0.035; .87 | -0.066; .88 | -0.127; .77 | | -0.009; .97 | | 0.199; .68 | | 0.208; .65 | | 0.244; .46 | | 0.802; .32 | | 0.558; .36 |
| Height (cm) | 1.335 | 1.482 | 1.021 | -0.286; .08 | 0.192; .70 | 0.474; .36 | | -0.253; .13 | | 0.330; .55 | | 0.583; .28 | | 0.232; .41 | | -0.164; .85 | | -0.396; .65 |
| BMI (kg/m^2^) | 0.682 | 0.538 | 0.662 | 0.147; .09 | -0.009; .93 | -0.166; .05 | | 0.173; .05 | | 0.004; .97 | | -0.170; .06 | | 0.126; .38 | | 0.191; .24 | | 0.066; .57 |
| Sitting height (cm) | 1.893 | 2.112 | -0.215 | -0.210; .59 | -0.309; .53 | -0.066; .87 | | 0.388; .36 | | 0.487; .40 | | 0.099; .82 | | 0.751; .27 | | 1.360; .15 | | 0.609; .38 |
| Sum of 3 skinfolds (mm) | 6.979 | 5.908 | 6.377 | 1.321; .24 | 0.877; .44 | -0.768; .32 | | 1.104; .32 | | 0.850; .47 | | -0.254; .74 | | 1.656; .36 | | 1.653; .39 | | -0.003; .99 |
| Corrected arm girth (cm) | 0.012 | 0.055 | -0.013 | -0.039; .70 | -0.004; .97 | 0.043; .64 | | -0.024; .80 | | 0.000; .10 | | 0.024; .80 | | -0.023; .88 | | 0.131; .48 | | 0.154; .31 |
| Corrected thigh girth (cm) | -0.252 | 0.218 | -0.443 | -0.475; .10 | 0.113; .73 | 0.442; .06 | | -0.521; .07 | | -0.011; .97 | | 0.509; .04 | | -0.900; .06 | | 0.101; .86 | | 1.000; .01 |
| Corrected calf girth (cm) | 0.200 | -0.009 | -0.021 | 0.207; .39 | 0.145; .55 | 0.010; .92 | | 0.251; .30 | | 0.213; .40 | | -0.037; .70 | | 0.143; .72 | | 0.350; .40 | | 0.208; .19 |
| Waist girth (cm) | 0.792 | 0.599 | 0.470 | 0.152; .59 | 0.345; .32 | 0.160; .56 | | 0.214; .45 | | 0.207; .57 | | -0.007; .98 | | 0.736; .11 | | 1.237; .06 | | 0.501; .10 |
| Hips girth (cm) | 2.840 | 2.507 | 2.248 | 0.336; .23 | 0.587; .09 | 0.099; .70 | | 0.394; .16 | | 0.597; .10 | | 0.202; .45 | | 0.615; .18 | | 1.184; .07 | | 0.569; .18 |
| Waist/hip ratio | -0.017 | -0.016 | -0.015 | -0.002; .52 | -0.001; .74 | 0.001; .77 | | -0.002; .51 | | -0.003; .42 | | -0.002; .63 | | 0.003; .52 | | 0.004; .58 | | 0.002; .64 |
| Muscle mass (kg) | -0.473 | -0.247 | -0.540 | -0.230; .16 | 0.041; .83 | 0.196; .15 | | -0.276; .09 | | -0.064; .75 | | 0.211; .13 | | -0.350; .18 | | 0.021; .95 | | 0.372; .10 |
| Fat mass (%) | 2.630 | 2.414 | 2.415 | 0.212; .62 | 0.297; .48 | -0.093; .74 | | 0.192; .65 | | 0.312; .46 | | 0.120; .67 | | 0.366; .59 | | 0.235; .74 | | -0.131; .77 |
| VO2 max. | -0.734 | -0.359 | -1.044 | -0.504; .16 | -0.313; .52 | 0.256; .59 | | -0.745; .12 | | -0.195; .70 | | 0.550; .25 | | -0.317; .59 | | 0.932; .25 | | 1.249; .11 |
| CMJ (cm) | -0.576 | 0.189 | -1.368 | 0.995; .05 | 0.726; .27 | -0.376; .47 | | 0.829; .11 | | 0.233; .73 | | -0.595; .25 | | 0.912; .28 | | 0.591; .59 | | -0.322; .71 |
| Curl-up | -0.478 | 1.773 | 0.730 | -1.806; .17 | -1.638; .16 | 0.198; .87 | | -2.681; .04 | | -1.998; .10 | | 0.683; .57 | | -2.941; .17 | | -3.840; .05 | | -0.899; .64 |
| Push-up | -0.844 | 0.101 | -0.433 | -1.105; .14 | -1.033; .15 | -0.122; .89 | | -0.836; .27 | | -0.836; .23 | | 0.000; 1.00 | | 0.072; .95 | | -1.134; .31 | | -1.205; .40 |

EG: Experimental group; CG: Control group.
